# Parasites in kelp‐forest food webs increase food‐chain length, complexity, and specialization, but reduce connectance

**DOI:** 10.1002/ecm.1506

**Published:** 2022-03-07

**Authors:** Dana N. Morton, Kevin D. Lafferty

**Affiliations:** ^1^ Department of Ecology, Evolution, and Marine Biology University of California Santa Barbara California USA; ^2^ Marine Science Institute University of California Santa Barbara California USA; ^3^ U.S. Geological Survey, Western Ecological Research Center, at Marine Science Institute University of California Santa Barbara California USA

**Keywords:** connectance, Copepoda, food webs, kelp forests, Macrocystis, parasite ecology, paratenic host, trophic transmission

## Abstract

We explored whether parasites are important in kelp forests by examining their effects on a high‐quality, high‐resolution kelp‐forest food web. After controlling for generic effects of network size, parasites affected kelp‐forest food web structure in some ways consistent with other systems. Parasites increased the trophic span of the web, increasing top predator vulnerability and the longest chain length. Unique links associated with parasites, such as concomitant predation (consumption of parasites along with their hosts by predators) increased the frequency of network motifs involving mutual consumption and decreased niche contiguity of free‐living species. However, parasites also affected kelp‐forest food web structure in ways not seen in other systems. Kelp‐forest parasites are richer and more specialized than other systems. As a result, parasites reduced diet generality and decreased connectance in the kelp forest. Although mutual consumption motifs increased in frequency, this motif type was still a small fraction of all possible motifs, so their increase in frequency was not enough to compensate for the decrease in connectance caused by adding many specialist parasite species.

## INTRODUCTION

**FIGURE 1:**
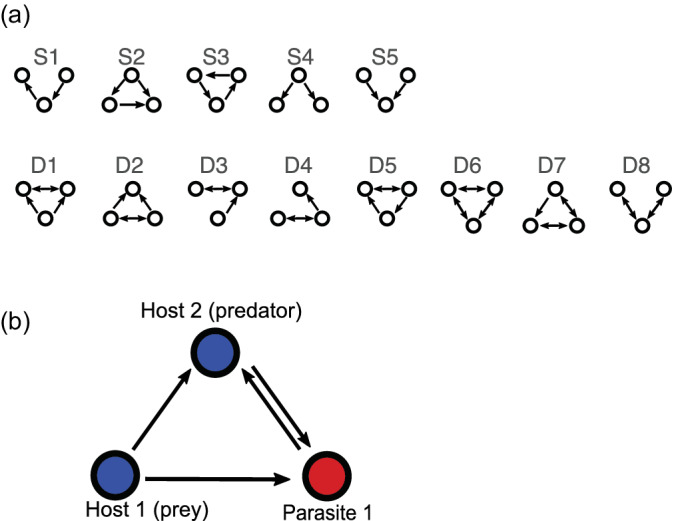
Food web motifs (McLaughlin, 2018; Milo et al., 2002). (a) The 13 types of three‐node motifs possible (McLaughlin, 2018; Milo et al., 2002). S1–S5 lack mutual consumption, D1–D8 have mutual consumption (double motifs). (b) Example of a double motif (D1) involving a parasitic interaction and intraguild predation. Parasite 1 is a parasite of Host 1 and Host 2. Host 2 is also a predator of Host 1, and Parasite 1 is killed via concomitant predation when Host 1 is consumed

Because parasite diversity derives from host diversity (Hechinger & Lafferty, 2005), it follows that, when metazoan parasites have been incorporated systematically into food webs, they alter network structure and function. Parasites typically increase species richness by about one‐third (Amundsen et al., 2009; Dunne et al., 2013; Lafferty et al., 2006; McLaughlin et al., 2020; Mouritsen & Poulin, 2002; Thompson et al., 2005), add biomass comparable to top predators (Kuris et al., 2008; McLaughlin, 2018; McLaughlin et al., 2020), and may participate in about 75% of links in some way (Lafferty et al., 2006). In addition to adding species, links, and biomass, parasites alter network structure in distinct ways (Lafferty et al., 2008). First, they add consumer pressure on free‐living species, increasing trophic level, food‐chain length and vulnerability (Table 1), particularly for top predators (Amundsen et al., 2009; Lafferty et al., 2006). Second, because parasites participate in most links, they can alter complexity metrics such as connectance (Table 1; Dunne et al., 2013; McLaughlin et al., 2020), degree distribution (Amundsen et al., 2009), and nestedness (Lafferty et al., 2006). Third, parasites that use a different host for each life stage (complex life cycle parasites) typically feed on phylogenetically distinct hosts throughout their lives (Parker et al., 2003), so parasite species have discontinuous feeding niches when life stages are aggregated into species nodes (Dunne et al., 2013). Lastly, parasites can alter a food web's subgraph structure (motifs; Milo et al., 2002), corresponding to ecologically relevant interactions among three or more species (Figure 1). Parasites often become prey when their host is killed by a predator (Johnson et al., 2010), so motifs involving mutual consumption become more common when parasites are included (Dunne et al., 2013; McLaughlin, 2018; McLaughlin et al., 2020). These changes to food‐web structure might also influence network stability.

**TABLE 1:** Definitions of metrics examined, summary of predictions, and observed outcomes

Prediction number	Metric or measure	Definition	Predicted direction	Prediction consistent with	Observed direction
1	Proportion parasite richness	Parasitic species richness/total species richness	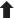	Dunne et al. (2013), Lafferty et al. (2006), Hechinger et al. (2011), Amundsen et al. (2009), Mouritsen and Poulin (2002), Thompson et al. (2005), McLaughlin (2018)	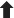
2	Proportion parasite links	Parasite–host links/all links, indicates parasite participation in the food web	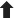	Amundsen et al. (2009), Hechinger et al. (2011), McLaughlin (2018)	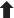
3	Chain length	Nodes in the longest food chain, indicates food‐web shape and maximum trophic level	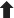	Amundsen et al. (2009), Dunne et al. (2013), Lafferty et al. (2006), McLaughlin (2018), Thompson et al. (2005)	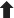
4	Link density	Links (*L*)/species (*S*), indicates how connected species are to the rest of the network	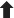	Amundsen et al. (2009), Dunne et al. (2013), Lafferty et al. (2006), McLaughlin (2018),	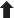
4	Connectance	*L*/*S* ^2^ observed links/possible links, describes overall network connectivity, linked to stability	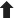	Amundsen et al. (2009), Dunne et al. (2013), Lafferty et al. (2006), McLaughlin (2018)	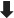
5	Vulnerability (free‐living)	Predators per resource, the diversity of predation threats	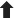	Amundsen et al. (2009)	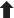
6	Vulnerability (all)	Consumers per resource, the diversity of threats from natural enemies	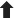	Amundsen et al. (2009), Dunne et al. (2013), Lafferty et al. (2006), McLaughlin, (2018), Thompson et al. (2005)	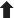
7	Generality	Resources per consumer, diet breadth	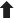	Marcogliese, (1996), Køie (1993), Palm and Caira (2008)	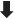
8	Most general species	The species with the highest generality, indicates species that are hubs of consumption	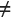	Marcogliese, (1996), Køie (1993), Palm and Caira (2008)	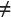
9	Degree distribution	The distribution (e.g., mean and SD) of link densities among species; shows the balance of highly connected (hubs) to weakly connected species	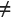	Lafferty et al. (2008), Warren et al. (2010)	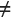
10	Niche contiguity	The similarity in trophic positions among diet items	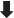	Dunne et al. (2013)	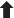
11	Frequency of intraguild predation (mutual consumption motifs)	Proportions of various rare three‐species interactions (e.g., a predator that eats a competitor)	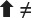	Dunne et al. (2013), McLaughlin (2018)	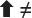

*Notes*: Metrics were selected based on examination in previous studies as well as their utility in describing network structure. Predicted or observed direction refers to the change in the metric in the food web with parasites added relative to the free‐living food web: ↑, increasing; ↓, decreasing, ≠, no change.

Changes to food webs following parasite addition could be either due to adding parasites, or just making food webs bigger (Dunne et al., 2013). Studies of “scale dependence” of food web properties have examined relatively small food webs (Martinez & Lawton, 1995), so whether these properties translate to much larger food webs is unknown. Link density (the number of links per species) tends to increase with network size (Banašek‐Richter et al., 2006; Hall & Raffaelli, 1993; Martinez, 1993, 1994; Schoener, 1989), possibly due to increased opportunities for interactions in larger webs (Warren, 1990). In contrast, connectance may decrease with network size in general, because the number of possible links often increases more quickly than the number of realized links (Banašek‐Richter et al., 2006; Dunne, 2006; Reide et al., 2010). For such reasons, increasing variability of degree distribution (the probability distribution of the number of connections each node has to other nodes) after adding parasites was explainable by increasing species richness, suggesting that parasites and predators affect degree distribution similarly (Dunne et al., 2013). For this reason, when comparing network metrics with and without parasites, it is helpful to distinguish between how food webs respond to adding parasite species versus adding species in general (Dunne et al., 2013).

Parasites have been systematically added to eight food webs using similar methodology (reviewed in McLaughlin et al., 2020). These webs were constructed for estuarine and soft‐sediment intertidal systems, as well as an Arctic lake, all characterized by relatively low diversity and low structural complexity, so it is unclear whether observed roles of parasites are specific to these types of systems. Giant kelp (*Macrocystis pyrifera*) forests differ from these habitats in some key ways, presenting an ideal system to examine whether the role of parasites varies among ecosystems. Specifically, kelp forests receive inputs from surrounding ecosystems (Zuercher & Galloway, 2019), create a dynamic three‐dimensional habitat (Dayton & Tegner, 1984; Ebeling et al., 1985; Edwards, 2004; Rogers‐Bennett & Catton, 2019), and support high species diversity and biomass (Balch & Scheibling, 2000; Carr & Reed, 2016; Schiel & Foster, 2015). Arctic lake and soft‐sediment intertidal habitats are relatively discrete, structurally simple, and species poor. Kelp forests contain more top predators and certain taxa (e.g., marine mammals, sharks) that are uncommon in salt marshes, lakes, or mudflats. Moreover, the kelp‐forest food web (Morton et al., 2021b) is more species rich than other currently published food webs that include parasites (Dunne et al., 2013; McLaughlin et al., 2020) and therefore presents new opportunities to understand the role parasites play in food webs.

Kelp forests along the coast of southern California (Point Conception to San Diego) have been studied more than anywhere else in the world (Carr & Reed, 2016; Dayton, 1985; Kushner et al., 2013). In this research effort, three food webs and a links database have been published for California kelp forests, but they are aggregated for certain taxonomic groups, have uneven levels of resolution, and do not include parasites (Beas‐Luna et al., 2014; Byrnes et al., 2011; Graham, 2004; Graham et al., 2008). The recently published kelp‐forest food web by Morton et al. (2021b) systematically resolves the free‐living and parasitic species that dominate biodiversity in this system, and therefore presents new opportunities to understand the kelp‐forest ecosystem and the role parasites play within it. To better assess whether parasites affect food webs due to increases in network size, or due to differences between parasites and hosts, we compared several network properties, before and after adding parasites (Morton et al., 2021b). Doing so expands several generalities about parasites and food webs, and points to exceptions and new perspectives.

### Hypotheses and predictions

We predicted that adding parasites might have similar effects on the kelp‐forest food web as on other food webs (Dunne et al., 2013; Lafferty et al., 2006; McLaughlin et al., 2020), except that (1) effects might be greater due to there being a higher proportion of parasite species in the kelp‐forest food web (Morton et al., 2021b) than other food webs with parasites, and (2) scale‐dependent effects might differ because the kelp‐forest food web is over 200% larger (in terms of total species) than past food webs with parasites. We also noted that some differences might be expected based on the different types of parasite life cycles seen in kelp forests. We did not test predictions about parasite biomass density because we did not have body sizes and densities for many taxa in this large network, nor did we assess robustness, as this requires separate methods for simulating disassembly (Chen et al., 2011; Lafferty, 2012; Lafferty & Kuris, 2009; Rudolf & Lafferty, 2011) and is therefore treated as a separate study. Specifically, based on past studies, we predicted that adding parasites to the kelp‐forest food web would (see Table 1 for summary):

1. Increase species and phyla richness at least as much (proportionally) as in other food webs with parasites (Amundsen et al., 2009; Dunne et al., 2013; Hechinger et al., 2011; Lafferty et al., 2006; McLaughlin, 2018; Mouritsen & Poulin, 2002; Thompson et al., 2005).
2. Increase consumer–resource links at least as much (proportionally) as in other food webs with parasites due to the addition of many host–parasite and predator–parasite links (Amundsen et al., 2009; Hechinger et al., 2011; McLaughlin, 2018).
3. Increase chain length, because even top predators are susceptible to parasites (Amundsen et al., 2009; Dunne et al., 2013; Lafferty et al., 2006; McLaughlin, 2018; Thompson et al., 2005).
4. Increase link density and connectance, especially when concomitant predation is considered, because parasites can be generalist and concomitant predation adds many links to food webs (Amundsen et al., 2009; Dunne et al., 2013; Lafferty et al., 2006; McLaughlin, 2018).
5. Increase free‐living vulnerability, especially for higher trophic levels, because top predators would have consumers (Amundsen et al., 2009; Lafferty et al., 2006).
6. Increase overall vulnerability, because concomitant predation can create many links to parasites (Amundsen et al., 2009; Dunne et al., 2013; Lafferty et al., 2006; McLaughlin, 2018; Thompson et al., 2005).
7. Assuming complex life cycles are common, increase generality even after accounting for network size, because host use by parasites with complex life cycles can be broader than predator diets, particularly for generalist parasites using paratenic hosts (an intermediate host in which no parasite development occurs, that may or may not be necessary in the parasite's life cycle; Køie, 1993; Marcogliese, 1996; Palm & Caira, 2008).
8. Change the ranking of the most general species in the food web due to adding generalist parasites, such as those using paratenic hosts (Køie, 1993; Marcogliese, 1996; Palm & Caira, 2008).
9. Alter degree distributions (more than expected by increasing network size alone) due to their expected effects on generality and vulnerability and because some parasites are highly specialized, and others infect hosts with few predators (Lafferty et al., 2008; Warren et al., 2010).
10. Make trophic niches less contiguous (when life stages are aggregated to species nodes) due to parasites feeding on diverse host taxa throughout their life cycles and inclusion of concomitant links (Dunne et al., 2013).
11. Increase the frequency of intraguild predation when concomitant links are included, and alter the frequency of other types of three‐species interactions that include mutual consumption (motifs) (Dunne et al., 2013; McLaughlin, 2018).

## METHODS

The kelp‐forest food web we used for these analyses is resolved for free‐living and parasitic species (Morton et al., 2021b) and is available on Dryad (Morton et al., 2021a). Morton et al. (2021b) describe the food‐web construction in detail, but we describe it briefly here. This food web was constrained to rocky‐reef habitat (both the benthos and water column) within the depth range of giant kelp (*M. pyrifera*) in the Santa Barbara Channel, California, USA, including the four northern Channel Islands. The food web is thus a large “metaweb,” characterizing kelp‐forest meta‐communities in this region, rather than a site‐specific web. Lists of free‐living and parasitic species were compiled through systematic literature review, long‐term monitoring group data sets, and extensive field sampling. The parasite species list includes metazoan species that use kelp‐forest species as hosts for at least one stage in their life cycle, with the acknowledgement that bacterial, viral, fungal, and protozoan pathogens are potentially important. Similarly, host microbiomes were not included. The fully resolved food web was constructed with life stages (e.g., larva, adult) as nodes rather than species because trophic interactions change as species grow (excepting benthic diatoms, planktonic diatoms, dinoflagellates, foraminifera, free‐living nematodes, bacteria, free‐living ciliates, copepod nauplii, filamentous algae, and invertebrate eggs, which are aggregate nodes). Detritus was included in the food‐web as four nodes: carrion, drift macroalgae, small mixed origin (such as would be consumed by a deposit or suspension feeder, with the recognition that this alone is a complex system deserving further resolution) and dissolved organic material. To allow comparison with other published food webs, we aggregated life‐stage nodes to the taxonomic species level before calculating metrics. In the aggregation process, resulting duplicate links were removed. If a species possessed both free‐living and parasitic life stages, the species was categorized as a parasite, but any free‐living links were retained (so a species might have both predatory links and parasitic links).

Adding parasites to a web of predator–prey interactions creates two additional sub‐webs: parasite–host and predator–parasite (concomitant predation). The classification of nodes and links as parasitic followed the methods of other food webs (Amundsen et al., 2009; Dunne et al., 2013; Lafferty et al., 2006; McLaughlin, 2018) and the framework presented by Lafferty and Kuris (2002). Some parasitic stages have a minimal energetic impact on their host (e.g., paratenic cyst stages, adult tapeworms), but these stages were considered to be parasitic as long as the impact was negative and the parasite obtained energy from the host. Mutualists and commensals were not categorized as parasitic. Predator–prey, parasite–host, and predator–parasite links between nodes were assigned based on published records, direct observation by the authors, expert opinion, and logical inference (based on encounter likelihood and compatibility of the interaction; Combes, 2001). A fourth sub‐web, parasite–parasite (some parasites will attack and consume other parasites within the host), is possible but was not observed. Both the nodes and the links list include metadata on the justification for inclusion, confidence, locality, and references, as well as taxonomic relationships, habitat niche, and consumer strategies (Morton et al., 2021b).

Due to uneven sampling effort, it is easy to miss some parasite–host links, particularly those with low prevalence or in understudied hosts. For this reason, the food web also estimates the number of “false negative” links between parasites and hosts, allowing us to provide an upper bound for certain food‐web metrics. Morton et al., 2021b describes the method for estimating false negative links in detail. Briefly, they used generalized linear models to estimate link probabilities based on taxonomic information, habitat associations, host trophic level, potential for infective stages in the host diet, and sampling effort. Then, they projected link probability assuming high sampling effort across all hosts. Parasite–host links that were not observed with existing sampling effort, yet predicted to be likely under high sampling effort, were flagged as possible false negatives.

We filtered the nodes list to exclude any species that were flagged as “extinct” (the full nodes list includes species that are rare or locally extinct, but of special conservation interest). We compared three web versions: predator–prey only, predator–prey + parasite–host, and predator–prey + parasite–host + predator–parasite. For each food‐web version, we compared the contributions of parasite and free‐living species to network size and linkages.

To control for increased network size when assessing parasite addition, we measured the deviation in metrics between each web version and simulated food webs of the same size and connectance (Williams & Martinez, 2000). To create hypothetical food webs, we used the niche model (a one‐dimensional model of food web structure) in the *trophic* package (https://rdrr.io/github/jjborrelli/trophic/) to simulate 1000 networks with size (*S*) and connectance (*C*) matching the empirical food web (Williams & Martinez, 2000). The niche model allowed comparison with previous analyses (Dunne et al., 2013). For the webs including parasites, we used adjusted connectance (Lafferty et al., 2006) for niche model simulations. For each version, we calculated the model error (ME) for the metric in question, (the normalized difference between the simulated model's median value and the empirical value; Williams & Martinez, 2008). Empirical metrics with |ME| > 1 fall outside the most likely 95% of model values and indicate a statistically significant difference from model values. ME allows comparison between webs of different sizes because ME is a measure of how much a given metric varies in simulated networks of the same size and connectance. The ME's sign (negative or positive) indicates whether the metric is under‐ or overrepresented in the empirical web relative to 1000 simulated niche models. If two networks have a similar magnitude and sign of ME, this indicates they vary from niche model expectation in a similar way. If the webs with and without parasites deviated from the niche model in the same way, it suggested they were structurally similar in that trait, whereas if they differed from the niche model in different ways, they likely differed in that trait independent of their size. Because this approach has typically been used with webs <100 nodes and niche‐model fits decline with network size (Dunne et al., 2013; Vinagre et al., 2019; Williams & Martinez, 2008; Williams & Purves, 2011; Wood et al., 2015), the MEs were interpreted with this potential confounding factor in mind. All metrics (Table 2) and niche‐model simulations were calculated in R Version 3.6.2 (R Core Team, 2019) with packages *igraph* (Csardi & Nepusz, 2006), *NetIndices* (Kones et al., 2009), *Cheddar* (Hudson et al., 2013), and *trophic* (https://rdrr.io/github/jjborrelli/trophic/).

To examine parasite impacts on network structure and address predictions 1–4 and 9, we calculated 14 metrics that describe food‐web structure (Table 2). We did not make specific predictions about each metric but report them here as they are commonly reported metrics for describing food webs and should be informative to readers who wish to make comparisons with other food webs. We also report metrics for food‐web assemblies corrected for false negative links where appropriate. We calculated two measures for connectance (prediction 4). Connectance was calculated using default methods (links per area; *L*/*S* ^2^) and as adjusted connectance, where the denominator depends on the sub‐webs included (Lafferty et al., 2006). For the predator–prey + parasite–host web, the denominator was calculated as the total possible considering the only two sub‐webs allowed: number of free‐living species × (number of free‐living species + number of parasite species). The denominator for predator–prey + parasite–host + predator–parasite web was the total possible, minus missing parasite–parasite interactions: (number of all species)^2^ − (number of parasite species)^2^. Link density, connectance, mean degree, mean generality, and mean vulnerability do not vary within the niche model, but we compared model predictions of longest chain length (prediction 3), with empirical values. Trophic level was calculated as prey‐averaged trophic level, excluding predator–parasite links.

**TABLE 2:** Summary of web metrics for each web assembly

Assembly	Predator–prey	Predator–prey + parasite–host	Predator–prey + parasite–host (with false negatives)	Predator–prey + parasite–host + predator–parasite	Predator–prey + parasite–host + predator–parasite (with false negatives)
Nodes	485	918	918	918	918
Links	8477	11,213	11,863	20,031	20,681
Link density	17.48	12.21	12.92	21.82	22.53
Connectance	0.036	0.013	0.014	0.024	0.025
Adjusted connectance^a^	–	0.025	0.027	0.030	0.032
Longest chain	9	10	–	7	–
Mean degree	36.59	25.64	–	46.56	–
SD degree	31.59	35.64	–	61.61	–
Mean generality	18.29	12.82	–	23.28	–
SD generality	26.02	23.06	–	51.59	–
Mean vulnerability	18.29	12.82	–	23.28	–
SD vulnerability	20.02	21.21	–	25.24	–
Minimum sum diet gaps	32,463	57,534	–	91,323	–

a Adjusted connectance calculated using the method of Lafferty et al. (2006). Denominator for predator–prey + parasite–host web was: number of free‐living species × (number of free‐living species + number of parasite species). Denominator for predator–prey + parasite–host + predator–parasite web was (number of all species)^2^ − (number of parasite species)^2^. False negative estimation described in Morton et al. (2021b).

Predictions 5–9 pertained to elements of degree (generality and vulnerability). To address predictions 5 and 6, we compared species vulnerability to both free‐living and parasitic enemies in the food web with and without parasites. Free‐living species are vulnerable to free‐living enemies (predators) and parasites, whereas parasites in this food web are only vulnerable to predation in the form of concomitant mortality (when their host is consumed by a predator). We tabulated the number of free‐living and parasitic enemies for free‐living species, and the number of enemies for parasites (concomitant links). We used a Wilcoxon rank sum test to determine whether these vulnerability distributions differed between free‐living consumers and parasites (JMP Pro V14, SAS Institute, Cary, North Carolina, USA). To address predictions 7 and 8, we compared consumer generality of free‐living versus parasitic species (diet breadth, excluding concomitant mortality). We tabulated the number of prey items (for predators) and hosts (for parasites), and we used a Wilcoxon Rank Sum test to determine whether the generality distributions differed between free‐living consumers and parasites (JMP Pro V14). To better understand how adding parasites affected qualitative differences in consumers, we compared the identities of the top 10 most general consumers with and without parasites. We then compared niche model predictions of measures of degree distribution (SD degree, SD generality, SD vulnerability) with empirical metrics to address prediction 9.

To assess contiguity of feeding niches (prediction 10), we determined the minimum number of diet gaps in the food webs using a simulated annealing method (Stouffer et al., 2006) employed in the *Cheddar* package (Hudson et al., 2013) R version 3.6.2 (R Core Team, 2019). We calculated the minimum sum of diet gaps for each empirical web using 10 optimizations each and took the mean. The lower the number of diet gaps, the more contiguous the feeding niches are. The variance was low for the optimizations and the overall trends for empirical webs using up to 100 optimizations did not change from results with 10. Because these optimizations were repeated on thousands of large‐simulated networks (1000 niche model simulations per version), we opted to use the minimum number of optimizations to balance computing time. Using the simulated annealing method (Stouffer et al., 2006), we reordered the empirical network matrices and simulated networks to minimize diet gaps, providing a visual representation of “trophic niche structure.” We tabulated the minimum sum of diet gaps for each network and compared the model predictions for niche continuity with empirical networks.

In previous studies, adding parasites to food webs changed the proportions of motifs that represent mutual consumption (“double motifs,” Figure 1), such as intraguild predation (Dunne et al., 2013; McLaughlin, 2018). To address prediction 11, we assessed the frequency of each type of double motif in each web version and simulated networks using the triad_census function in *igraph* (Csardi & Nepusz, 2006), R version 3.6.2 (R Core Team, 2019), and compared model predictions of double motif proportions with empirical proportions.

## RESULTS

### Prediction 1: Proportion of parasite richness

**FIGURE 2:**
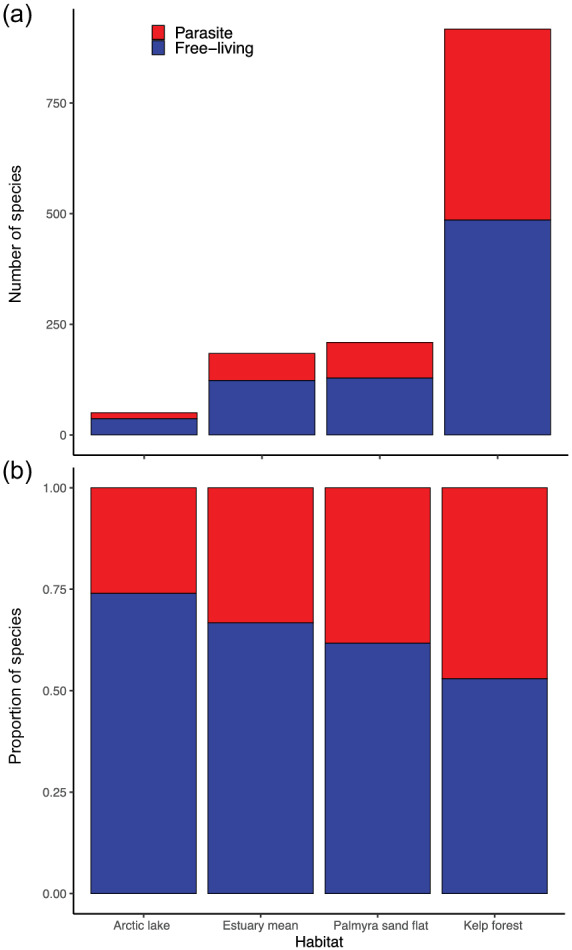
Parasite and free‐living richness in the kelp‐forest food web relative to other published food webs with parasites that used similar methods (Amundsen et al., 2009; Dunne et al., 2013; Lafferty et al., 2006; McLaughlin, 2018). Panel (a) shows total species richness, (b) shows proportions of parasites versus free‐living species

Parasites contributed more species richness to the kelp forest (absolutely and proportionally) than to any other food web, in line with our first prediction (Figure 2a, Appendix S1: Figure S1). The predator–prey web had 485 free‐living species (compared with 22–140 species in past parasite food webs), and parasites added another 433 species (47% of species; Table 2 and Figure 2a; Appendix S1: Figure S1). Parasites added three additional phyla to the web. Several phyla containing parasites were already represented by the free‐living invertebrates (e.g., Nematoda, Arthropoda), but there were two phyla that did not have any free‐living representatives (Acanthocephala, Dicyemida).

### Prediction 2: Proportion of parasite links

**FIGURE 3:**
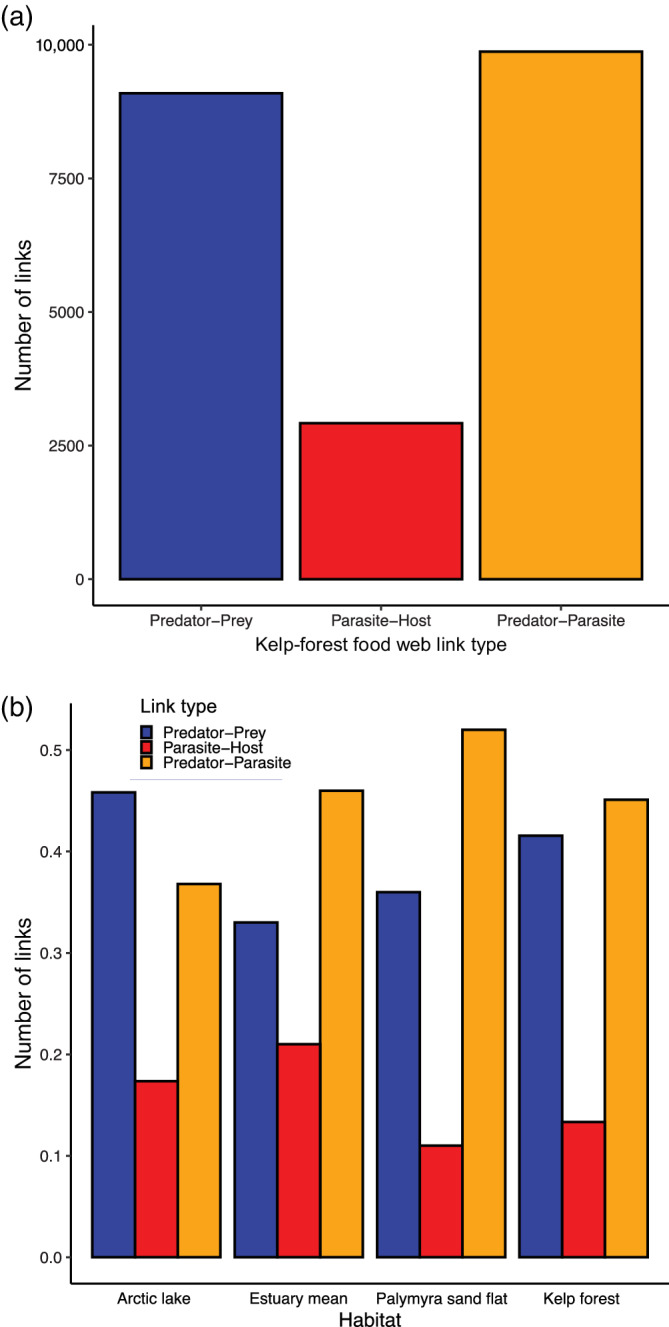
(a) Number of links in each sub‐web of the kelp‐forest food web (aggregated to species) and (b) proportions of links in each sub‐web web relative to other published food webs with parasites that used similar methodologies (Amundsen et al., 2009; Dunne et al., 2013; Lafferty et al., 2006; McLaughlin, 2018)

Contributions of parasites to the total link count (Figure 3a) in the food web were similar to contributions in other published webs (prediction 2; Figure 3b). Each predator–prey link creates an opportunity for either trophic transmission or concomitant mortality of parasites inhabiting the prey species, depending on the types of parasites and whether the predator is a compatible host. In this study, predator–parasite links represent only concomitant mortality of parasites. Any predator–prey links that lead to trophic transmission were not included in the predator–prey sub‐web (e.g., not double counted). The predator–prey sub‐web contained 42.3% of links, 13.6% of links were parasite–host links, and 44.2% of links were between predators and parasites (Figure 3a). Trophically transmitted parasite–host links made up 10.3% of all links in the food web and 75.7% of all host–parasite links. These proportions were within the ranges of proportions observed in other published webs (Figure 3b).

### Prediction 3: Chain length

Longest chain length was also sensitive to link type. The longest chain was one link longer in the web with parasites without concomitant links and this difference was greater than expected based on network size (Tables 2 and 3). Both of these web versions had somewhat longer chains than predicted by network size, but the web with parasites deviated from the niche model by twice as much (Table 3). Surprisingly, longest chain length was significantly lower in the web with parasites and concomitant links than in the predator–prey web, and was similar to expectations based on network size (Tables 2 and 3). Aggregating life stages to species meant that predators of individual life stages became predators of the entire species, which shortened some food chains. This reinforces the idea that the effects of parasites on network‐level structures depend on the types of parasite links considered.

**TABLE 3:** Model errors (ME) for the metrics that vary within the niche model (Williams & Martinez, 2008)

Assembly	Predator–prey	Predator–prey + parasite–host	Predator–prey + parasite–host + predator–parasite
Longest chain	**−1.50**	**−3.00**	0.00
SD degree	**−3.33**	−2.65	**−8.86**
SD generality	0.75	2.75	**3.50**
SD vulnerability	**−7.65**	−4.25	**−14.69**
Minimum sum diet gap	**−16.26**	−8.78	**−16.67**

*Notes*: |ME| > 1 indicates that empirical metrics were significantly different from model predictions after standardizing for web size. Positive MEs indicate a metric was greater than predicted in the empirical food web. If empirical webs have MEs that differ by >1, this indicates that the metric varies between the webs.

### Prediction 4: Link density and connectance

**FIGURE 4:**
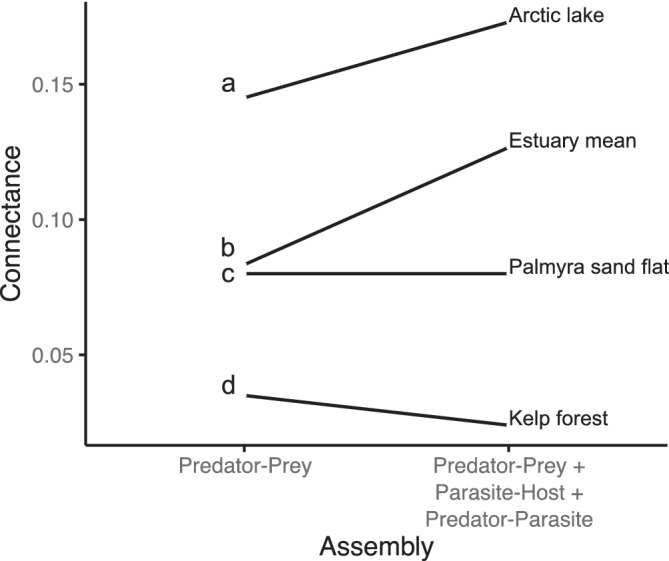
Trends in connectance with inclusion of parasites the food webs (a) Arctic lake, (Amundsen et al., 2009), (b) estuary mean (Dunne et al., 2013; Lafferty et al., 2006), (c) Palmyra atoll sand flat (McLaughlin, 2018), and (d) kelp forest (present study). Unadjusted connectance (defined in Table 1) is shown

Link density (*L/S*) slightly increased when parasites were included with concomitant links, in line with prediction 4 (Table 2). Even though adding parasites increased network size by 88.5%, link density decreased from 17.5 links per species to 12.2 links per species when only the host–parasite sub‐web was added. When concomitant links were included, link density increased by 25.3% relative to the free‐living web. However, adding parasites to the food web did not increase connectance as seen in other food webs (Figure 4). At 3.6%, connectance in the free‐living kelp‐forest food web was lower than in most food webs (Figure 4, Dunne et al., 2004, 2013; McLaughlin, 2018; Reide et al., 2010), possibly due to its large size and high resolution (Dunne et al., 2004). When parasites were added with concomitant links, connectance decreased to 2.4% (unadjusted), or 3% (adjusted; Table 2). When parasites were added without concomitant links, connectance decreased to 1.3% (unadjusted), or 2.5% (adjusted; Table 2).

### Prediction 5: Free‐living vulnerability

**FIGURE 5:**
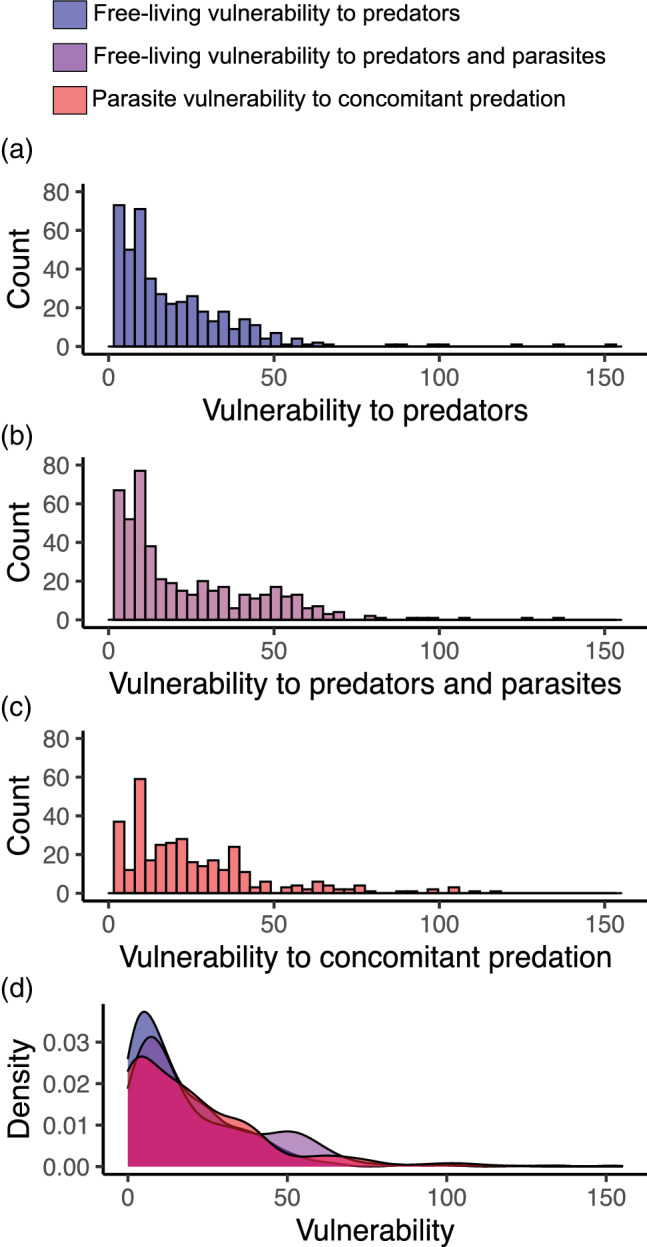
Vulnerability of free‐living species and parasites to enemies. Panels (a–c) are histograms: (a) free‐living vulnerability to predators, (b) free‐living vulnerability to both predators and parasites, (c) parasite vulnerability to concomitant predation. (d) The same information as density plots, overlayed for easier comparison. Terms are defined in Table 1

**FIGURE 6:**
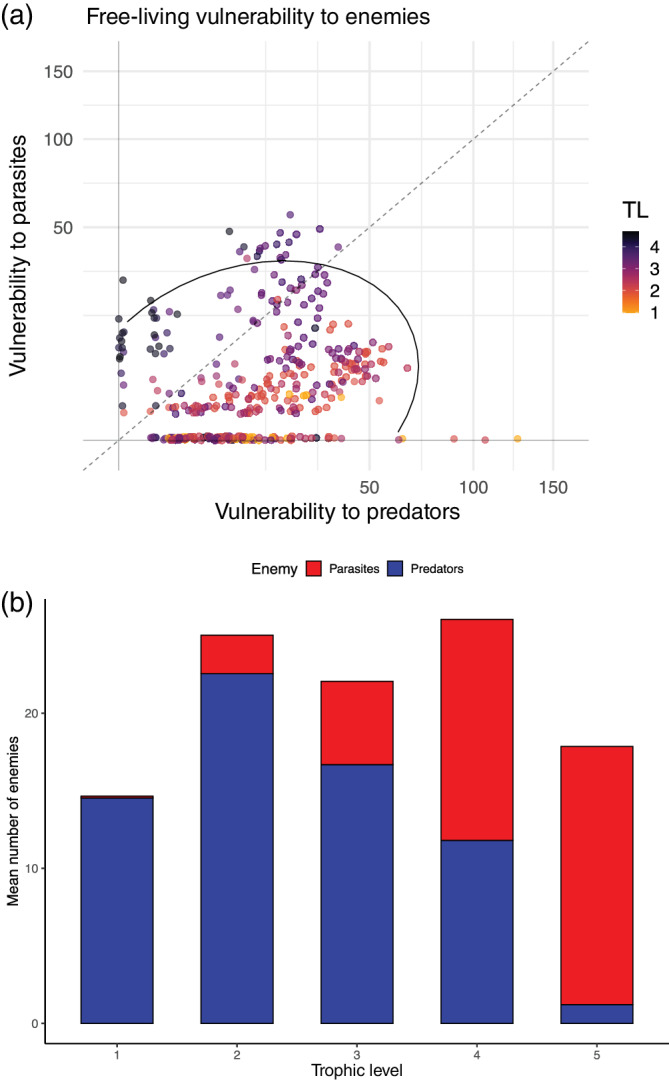
Vulnerability by enemy type. (a) Vulnerability to parasites versus vulnerability to predators, color‐coded by trophic level (TL). The dashed 1:1 line represents equal vulnerability to parasites and predators; the ellipse represents 95% of the observations assuming a multivariate normal distribution. Most species had either relatively few natural enemies, many predators, or many parasites, and only mid‐level predators had many predator and parasite species. (b) Mean enemies per free‐living species (same data as panel a but binned by trophic level). Middle trophic levels (2–4) were most vulnerable overall. The average plant and grazer had very few parasites, whereas and the average top predator had many parasites and few predators

Adding parasites increased free‐living vulnerability (Figure 5a,b, *Z* = 5.329, *p* < 0.0001) in line with prediction 5, whereas parasites were less vulnerable overall than free‐living species (Figure 5c, *Z* = −2.499, *p* < 0.0125), possibly because, when a parasite's host is eaten, this sometimes transmits the parasite to another host (and such predator–parasite links are not considered as losses). Consistent with other food webs, adding parasites increased vulnerability of mid to upper trophic levels (Figure 6). Most species tended to have more predators than parasites, and most species with many enemies had either many predators or many parasites. Two species had >100 predator species, only two hosts had ≥50 parasite species. Only species of intermediate trophic levels had many predator and parasite species. The average plant and grazer had very few parasites as defined in this study (metazoans), approximately half of free‐living species (mostly primary producers) had no parasites recorded from them, whereas the average top predator had many parasites and few predators.

### Prediction 6: Parasite vulnerability

Even though parasites were less vulnerable to enemies than free‐living consumers, adding parasites increased network mean vulnerability (all consumptive links, including concomitant) relative to the free‐living web (Table 2). Concomitant links were more common than predator–prey links (Figure 3) and are a unique type of trophic interaction. The inclusion of these links increased overall food‐web vulnerability.

### Prediction 7: Generality

**FIGURE 7:**
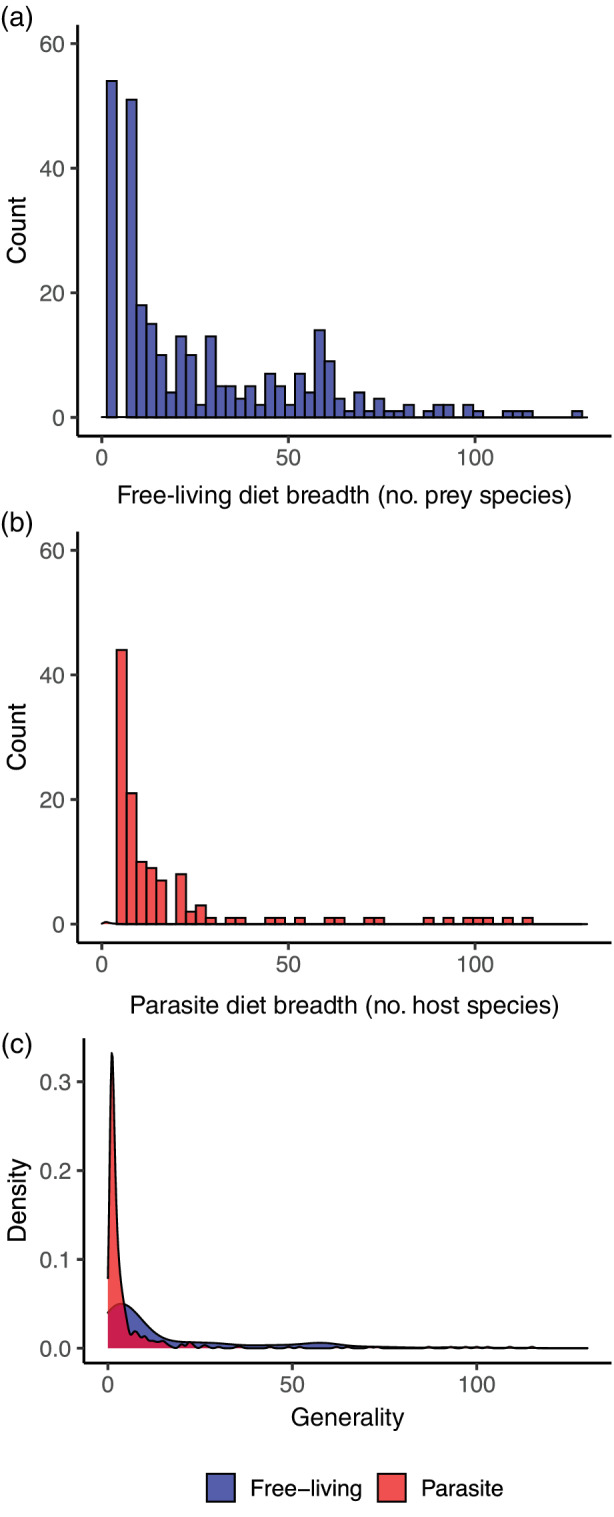
Diet breadth (generality, defined in Table 1) of free‐living versus parasitic species in food web containing predator–prey and parasite–host links. (a) Diet breadth of free‐living species as counts of prey species, (b) diet breadth of parasites, as counts of host species, (c) density plots of diet breadth (generality), overlayed for easier comparison. Concomitant links are not included in counts of prey species

In contrast to prediction 7, mean generality was lower in the predator–prey + parasite–host web (Table 2). Parasites had lower diet breadth overall than free‐living species (Figure 7, *Z* = −9.724, *p* < 0.0001). The median diet breadth was 7 for free‐living predators (IQR 4–29) and 2 for parasites (IQR 1–4). Many parasites did have complex life cycles and broad host ranges at the species level, but a large proportion of parasite species did not (e.g., parasitic copepods), or not all life stages were present in the food web. Most parasites were more specialized than free‐living taxa, which also reduced overall generality (Figure 7).

### Prediction 8: Most general species

**FIGURE 8:**
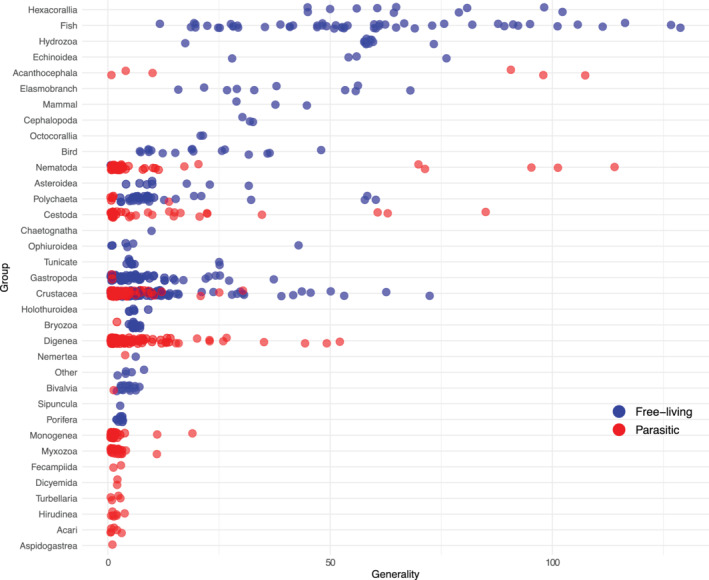
Diet breadth of free‐living and parasitic species by organismal group in the food web containing predator–prey and parasite–host links

Although predators had broader diets than parasites overall, the generality distributions overlapped: the most generalist parasite had 114 hosts, and the most generalist predator had 133 prey. Of free‐living species, anemones (Hexacorallia), fishes, elasmobranchs, and birds had the broadest diets (Figure 8). Sponges, bivalves, and other filter feeders had the lowest diet breadth, but this was, in part, due to aggregating many phytoplankton species. The most general parasitic groups (at the species level) were acanthocephalans, nematodes, and cestodes, which are all trophically transmitted parasites that use paratenic hosts in their life cycles. As a result, the 10 most generalist taxa changed when parasites were included (Table 4). In the free‐living web, the most general consumers were fishes and anemones. Three fish parasites joined the generalist ranking. The fourth and seventh most general species were seal parasites (*Pseudoterranova decipiens* and *Corynosoma strumosum*) that use fishes as intermediate hosts, and the 10th most general was *Hysterothylacium aduncum*, a nematode that uses fishes as its final host. Therefore, the parasite community had many specialists and a few extreme generalists.

**TABLE 4:** Top 10 most general species by web assembly

	Predator–prey		Predator–prey + parasite–host	
Rank	Taxon	Diet breadth	Taxon	Diet breadth
1	*Semicossyphus pulcher* (F)	133	*Semicossyphus pulcher* (F)	133
2	*Embiotoca jacksoni* (F)	128	*Embiotoca jacksoni* (F)	128
3	*Anisotremus davidsonii* (F)	116	*Anisotremus davidsonii* (F)	116
4	*Halichoeres semicinctus* (F)	114	***Pseudoterranova decipiens*** **(P)**	114
5	*Paralabrax clathratus* (F)	109	*Halichoeres semicinctus* (F)	114
6	*Urticina lofotensis* (A)	102	*Paralabrax clathratus* (F)	109
7	*Phanerodon furcatus* (F)	101	***Corynosoma strumosum*** **(P)**	107
8	*Hypsypops rubicundus* (F)	99	*Urticina lofotensis* (A)	102
9	*Anthopleura sola* (A)	98	*Phanerodon furcatus* (F)	101
10	*Caulolatilus princeps* (F)	92	***Hysterothylacium aduncum*** **(P)**	101

*Notes*: Parasitic species are shown in boldface type. A, anemone; F, fish; P, parasite. Diet breadth does not include concomitant links.

### Prediction 9: Degree distribution

Changes to the vulnerability distribution in the food web with parasites led to greater degree SD than predicted by network size (Table 3). The network‐level SD generality was nominally larger in this web than the predator–prey web, but significantly less than expected based on network size alone (Table 3). When parasites were included with predator–parasite links, generality SD was even lower than expected based on network size (Table 3).

### Prediction 10: Niche contiguity

**FIGURE 9:**
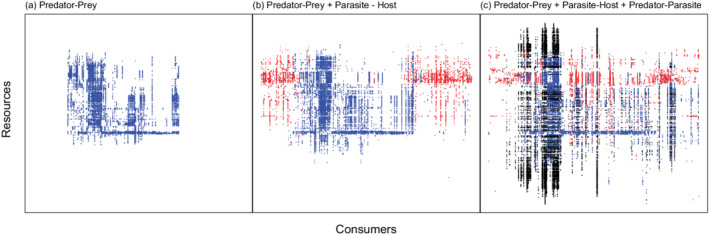
Graphical representation of each version of the food web as a matrix, with species ordered to minimize the number of gaps in the diet (simulated annealing method; Stouffer et al., 2006). Consumers are along the *x*‐axis, resources along the *y*‐axis. Matrices are identical in scale, but the order of species may vary among plots. Points indicate trophic links: blue are predator–prey (free‐living) interactions, red are parasite–host interactions, and black are predator–parasite interactions (cases where a predator consumed a parasite, leading to the death of the parasite). The vertical spread of points over a consumer indicates its feeding niche. (a) Predator‐prey web, (b) predator‐prey and parasite‐host web, and (c) predator‐prey, parasite‐host, and predator‐parasite web

In contrast to prediction 10, the predator–prey + parasite–host web had the most contiguous feeding niches (fewest diet gaps for the network size), whereas the other two web assemblies had fewer contiguous niches than predicted for their size and were similar (Table 3). When predator–parasite (concomitant) links were included, the trophic niches of free‐living consumers were less contiguous (Figure 9). Simply put, consumers ate more species when parasites were included, because they ingest parasites along with free‐living prey (as shown by comparing the spread of prey in Figures 9b vs. 9c). Concomitant links appeared at the edges of the niche range of consumers, increasing the total range and decreasing contiguity. However, given that parasites are rarely counted as food items in diet studies (due to a negligible energetic contribution), panel 9c should not be considered a diet niche, as much as a new network property.

### Prediction 11: Motif frequencies

**FIGURE 10:**
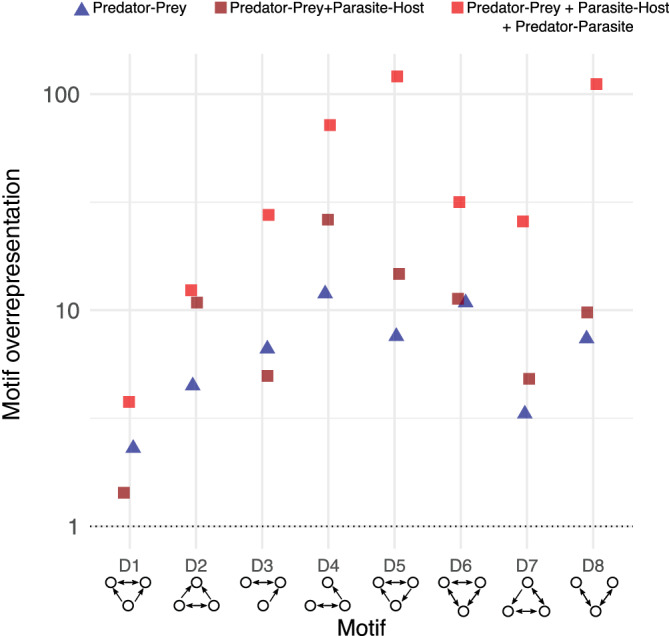
Standardized representation of double motif frequency in empirical food webs. Overrepresentation (*y*‐axis, log‐scale) calculated by comparing empirical frequencies to niche model predictions to control for the effect network size on motif frequencies and allow comparisons among empirical webs

Adding parasites greatly increased the frequency of three‐species interactions (motifs) that include mutual consumption (Figure 10, Appendix S1: Table S1). For all of these interactions except D1 (exploitative competition + intraguild consumption), the food web with concomitant (predator–parasite) links had a greater proportion of three‐species interactions involving mutual consumption than the predator–prey web or the web including parasites but not concomitant links. D4 (exploitative + mutual consumption), D5 (trophic loop + mutual consumption), and D8 (apparent competition + exploitative competition) were especially enhanced by the addition of concomitant links. Trophic loops (S3) were also much more frequent in the webs including parasites and concomitant links, and intraguild consumption (S2) was most frequent in the web including concomitant links. Adding parasites therefore changed the organization of the base structural units of the food web.

## DISCUSSION

### Species richness and food‐web size

The high parasite richness observed derives from the kelp‐forest data being a time‐integrated metaweb for the entire Santa Barbara Channel Region, rather than a single lake or estuary. Furthermore, the kelp‐forest web included more parasite–host and predator–prey records from the literature than did other food webs with parasites, which were constructed mostly from field sampling and inference (Amundsen et al., 2009; Dunne et al., 2013; Lafferty et al., 2006; McLaughlin, 2018). However, neither of these characteristics should have biased our sampling to overrepresent parasites. Instead, the sampling used to construct the food web was more likely to fail to detect parasite species than free‐living species, an effect that may become more pronounced as food webs grow. Heavily parasitized top predators are often challenging to collect (such as birds, mammals, and sharks, that are common in kelp forests), and cryptic parasite species are discovered regularly (Leung et al., 2009; Miura et al., 2005; Poulin, 2011; Soldánová et al., 2017), so the true parasite richness is likely higher than what was observed in sampling and reported in the literature. This may be why many unobserved parasite–host links had high probabilities of false negatives in the kelp‐forest food web (Morton et al., 2021b). Even though we report many parasites from the kelp‐forest food web, further parasitological studies will surely reveal more parasite species and even more links among hosts and parasites.

Another difference among published food webs is the extent that nodes are aggregated. For example, the estuary webs analyzed within Dunne et al. (2013) contain an aggregate node for “macroalgae” of which there are a few rare species, whereas the kelp‐forest food web has over 50 species of macroalgae, many of which are quite common. The estuarine webs have two categories for detritus (“carrion” and “detritus”), but the kelp‐forest web has four. These differences in web construction likely reflect system differences more than methodological ones.

Rather than methodological differences or bias, the high proportion of parasite richness observed in the kelp‐forest food web is likely due to the high host richness in this system, which derives from the more structurally complex, productive, and open nature of kelp forests. Kelp forests support many wide‐ranging sharks, marine mammals, and birds, which were host to groups of parasites that were less common and less diverse in other food webs, particularly acanthocephalans and shark tapeworms.

### Effects of adding parasites on connectance

Past work implied that parasites increased food web connectance (Dunne et al., 2002). The unexpected reduction in connectance in the kelp‐forest food web after adding parasites therefore challenges this generality. One reason for this difference might be how connectance scales with network size. As networks grow, the proportion of specialists tends to increase (Dunne et al., 2002). For instance, if we assume that new species added to the network had the same average link density as the free‐living species in the food web (17.48), a network of the same size as the food web with parasites (918 species) would have an even lower connectance than we observed (suggesting that, rather than decreasing connectance, parasites slowed the decline in connectance due to scaling).

Additionally, it is possible that decreasing connectance after adding parasites was due to false negative host–parasite links. Others have noted that, as networks grow, it becomes harder to sample all interactions (Goldwasser & Roughgarden, 1997; Hall & Raffaelli, 1993; Paine, 1988; Polis, 1991; Winemiller & Polis, 1996), and our large host–parasite network might have missed many rare host–parasite links. Indeed, correcting for statistically estimated false‐negative links would have increased the number of host–parasite links by 650, however link density would increase by only 0.7, and connectance would increase by only 0.1% (Table 1). For these reasons, we consider that the low connectance seen in this study is robust to sampling error and cryptic species.

Moreover, the unexpected reduction in connectance after adding parasites could be due to differences between kelp‐forest parasites and parasites from other systems. Parasite–parasite interactions in the kelp forest, or predation on parasite free‐living stages, increase connectance and are common in other food webs with parasites (Lafferty et al., 2006), yet none were observed in the kelp forest. Regardless, excluding parasite–parasite interactions from consideration as possible links would still mean that adding parasites reduced network connectance from 3.6% to 3% (as opposed to 2.4%). Similarly, the predator–prey + parasite–host web connectance were adjusted to account for the false negative potential predator–parasite and parasite–parasites links, so adding only host–parasite interactions reduced connectance from 3.6% to 2.5% (as opposed to 1.3% unadjusted; Lafferty et al., 2006). Regardless of how we calculated it, adding parasites decreased connectance to the kelp‐forest food web.

A final reason connectance declined in the kelp forest but not in other food webs was because kelp‐forest parasites overall were more specialized than in other systems. In estuaries, trematodes contribute to high connectance for four reasons: many trematode species infect a single host snail, these trematodes interact within the snail, many predators eat free‐swimming larval trematodes, and trematodes are often highly generalist in their second‐intermediate and final hosts (Lafferty et al., 2006). In the kelp forest, trematodes (Digenea, Aspidogastrea) tend to be more specialized. Instead, the kelp‐forest food web had many parasitic copepods, which are relatively more specialized and not trophically transmitted, so they are more weakly connected within the food web. Many parasitic copepods have a planktonic larval stage (or a larval stage that is parasitic on a planktonic organism), so the diversity of this group may be a unique feature of more open marine ecosystems. Comparisons with other subtidal food webs could assess the generality of these patterns.

Given that high host specificity among kelp‐forest parasites seems to reduce connectance, how might this affect kelp‐forest food‐web dynamics? A common expectation is that connectance increases the extent that perturbations flow through food webs (Romanuk et al., 2017); thus, the reduced connectance associated with parasitism might be assumed to buffer kelp forests from perturbations. However, given that parasites are rarely key resources for other consumers (even if they are eaten accidentally), specialist parasites are a dead end for perturbations. On the other hand, if specialization makes parasites even more sensitive to secondary extinctions than reported for other systems, this could make parasites particularly good indicators of host diversity in this system. Specialization could make host exploitation more effective, thereby increasing interaction strengths between parasites and the hosts they specialize on. Therefore, host specialization may create a network with efficient parasites that interact intensely with their hosts, but less intensely with other parasites. They seem unlikely to reduce perturbances. Rather they should reduce overall network robustness because specialists are particularly sensitive to reductions in host diversity (Lafferty, 2012).

### Predation on hosts affects network structure

Predators eat hosts and the parasites within them. Concomitant predation (when the predator digests the parasite) is not generally recorded in food web studies, yet it was the most common link type in this and other food webs with parasites (Lafferty et al., 2006). Because parasite consumption does not usually benefit a predator energetically, we often excluded concomitant links from topological measures (such as trophic level), or calculated measures with and without such links. However, concomitant links do affect parasites, as indicated through vulnerability. Counter to predictions, when concomitant links were included, the longest chain shortened. The chain in question shortened due to concomitant predation on parasite life stages that were aggregated to species, but when life stages were considered separately, parasites did increase chain lengths (Morton, 2020). The proportion of predators in the system that can serve as hosts versus those that will lead to the death of the parasite will determine parasite dynamics in the system. For example, larval acanthocephalans, tapeworms, and trematodes can modify host behavior to increase predation risk, and thus parasite transmission to the next host (Bethel & Holmes, 1977; Lafferty & Morris, 1996; Ness & Foster, 1999), but this may also enhance concomitant predation risk to non‐host species. We did not include enteric bacteria or protozoa in the food web but ingesting a prey's microbiome would also likely count as concomitant predation and such interactions could be included in future studies. Trophically transmitted parasitism was the most common type of parasite–host link (recall that trophically transmitted parasite links to hosts were only included in the parasite–host sub‐web and were not included in the predator–prey sub‐web, so no links were double counted). Parasite‐induced behavior can increase transfer of energy to upper trophic levels (Lafferty & Morris, 1996), thus the contribution of parasites to energy flow in kelp forests is an area for future work. Although concomitant links do not measure energy flow, they are key to understanding the interactions between predator and parasite populations in food webs (Johnson et al., 2010).

### Parasites alter vulnerability

As in other studies, adding parasites to the kelp‐forest food web increased vulnerability and variation in vulnerability. Unfortunately, vulnerability and degree variability may have been affected by decreasing niche model fit with web size (Dunne et al., 2013; Vinagre et al., 2019; Williams & Martinez, 2008; Williams & Purves, 2011; Wood et al., 2015), so it is difficult to distinguish whether these effects were parasite‐specific or due to network size. Unrelated to network size, chain length increased and vulnerability changed across trophic levels. Vulnerability of top predators increased more than that of primary consumers, and species at intermediate trophic levels became the most vulnerable to enemies overall. Top predators are subject to less predation, so their parasites were less vulnerable to concomitant predation. Adding parasites to the kelp‐forest food web increased vulnerability SD more than predicted by network size (Dunne, 2006). However, vulnerability SD tends to be underestimated by the niche model (Vinagre et al., 2019), so this could explain the relatively high |ME|s for this metric.

Primary producers had the fewest parasites, but this is partly a matter of defining a “plant parasite.” Most animal grazers do not eat the whole “plant” and feed on many different plants in their lives, so we considered these to be micropredators. We classified species that do not eat the whole plant but that live and feed on only one host individual to be true parasites, such as the kelp‐gribble isopod. Two species of amphipod (including kelp‐curling amphipods) and one snail appeared similar to parasites in that the plant provides habitat and is not completely eaten (Cerda et al., 2010; Coyer, 1984; Hobson & Chess, 1976) but these species move between hosts regularly so we considered these to be micropredators. If they were classed as parasites instead, they would have little effect on overall vulnerability and would not substantially change the average number of parasites feeding on the lowest trophic levels. Moreover, the food web does not include symbiotic microbial organisms, such as protozoans, viruses, bacteria, or fungi, but inclusion of these groups would almost certainly add parasites across all trophic levels.

### Parasites alter generality

Excluding parasites as diet items, parasites did not increase predator generality. Rather parasites affected network generality through their host ranges. Parasitic taxa in the kelp‐forest food web were more specialized than free‐living species (whether calculated at the stage or species level). This was driven by the most species‐rich groups: trematodes (Digenea) and copepods. Most parasitic copepods did not exhibit a broad host range throughout their life cycle. The high species richness of parasitic copepods was unique to the kelp‐forest food web and it reduced connectance and diet breadth. Kelp‐forest trematodes are relatively host‐specific at each stage and most do not use paratenic hosts, so their total host range is somewhat constrained. Furthermore, not all parasite species had all life stages in the kelp‐forest food web (e.g., adult trematodes found in transient hosts), so their host breadth within the kelp forest was narrower than in food webs that contain all trematode life stages. In estuary food webs, trematodes tend to complete most or all of their life cycle in the estuary and make up a substantial proportion of the links and biomass in these systems, so the role of trematodes differs between these ecosystems, but further elucidation of trematode life cycles may reveal additional participation in kelp forests.

Although there were many common specialist parasite species, the most generalist parasites were common in fish dissections, had low host‐specificity as larvae, and used paratenic hosts (Morton et al., 2021a). An acanthocephalan of pinnipeds and two nematodes (one of fish, one of pinnipeds) were among the most general consumers, along with iconic kelp‐forest fishes such as the California sheephead and kelp bass (and, in fact, parasitizing them as well). Larvae of shark tapeworms and seabird acanthocephalans were also abundant but were not quite as general. Although an individual cyst may not have a strong effect on a host, these stages accumulated in predators (Morton et al., 2021a) and may have impacts at high intensities. Additionally, these parasites have life cycles that are robust to reduced host diversity and paratenic hosts are important for transmission to final hosts. Trophically transmitted worms with more successive hosts tend to infect a greater diversity of host species and taxa, particularly in middle life stages, and the number of potential host species available constrains parasite generalism (Benesh et al., 2020). To our knowledge, this is the first food web with parasites that features paratenic host use as a prominent food‐web feature. In food‐web construction, likely paratenic hosts were counted as food‐web links, as determined through likelihood of encounter with the infective stage and host compatibility. Paratenic host–parasite links made up 4.3% of total links, 42.3% of trophically transmitted parasite links, and 25.5% of all host–parasite links. Exclusion of this link type would make parasites appear extremely specialized, further reducing food web connectance. Parasites using paratenic hosts had ample opportunities for hosts in the kelp forest and were among the most abundant parasites in dissections (Morton et al., 2021a). However, these extreme generalists were outnumbered by non‐trophically transmitted specialists in terms of species richness. Generality SD was less than predicted based on network size for the webs including parasites. This may be attributed to the wide distribution of parasite host breadth, which almost entirely overlapped with free‐living consumer diet breadth distribution.

### Parasite species have unique trophic niches and alter network motifs

Parasites affected the feeding niches for predators differently than found in past studies. Species‐rich groups such as parasitic copepods had a relatively narrow host range so they had the effect of increasing overall niche contiguity and decreasing niche range. Trematodes and other parasites with complex life cycles did not have the same effects on niche contiguity as in other food webs. The methods of assessing niche contiguity in our study differed from other food webs, which were assessed using body size to order feeding niches (Dunne et al., 2013). We did not have body size data for all species in the food web, so we used a simulated annealing method that performs ordinations of the food web matrix to determine arrangements with the fewest diet gaps (Stouffer et al., 2006). To compare the kelp‐forest food web to the estuary webs, we used the simulated annealing method to characterize niche contiguity of the estuary food webs (Appendix S1: Figures S2–S7) and results were similar to previous studies: parasites in estuary food webs made feeding niches less contiguous when parasites stages were aggregated to the species level because parasites with complex life cycles use a wide range of host types.

Inclusion of concomitant links led to an increase in frequency of apparent competition motifs that were rare in the free‐living food web, a structural change that is consistent with patterns in other food webs and not linked to increasing network size (Dunne et al., 2013). However, we downplay this difference because the accidental ingestion of a parasite does not contribute to a predator's energetic gain and is unlikely to have a strong effect on energy flow or stability. This is because accidental consumption of a parasite within a host, although (usually) bad for the parasite, usually has inconsequential benefits for the consumer. Not only do parasites alter motifs, but the meaning of a motif differs between parasites and free‐living consumers.

### Implications for stability

We did not examine kelp‐forest stability or robustness, but parasites reduced connectance in ways that likely do not increase stability, and parasites reduce overall food web robustness in other studies (Lafferty, 2012; Lafferty & Kuris, 2009; Rudolf & Lafferty, 2011), so we hypothesize that parasites would reduce kelp‐forest food web robustness as well. Adding parasites also increased vulnerability, especially for higher trophic levels, which could add stabilizing density‐dependence for consumers that otherwise lack top‐down regulation. These changes in trophic structure may also correspond to a change in trophic coherence, a network feature linked to stability (Johnson et al., 2014). We aim to explore how parasites affect kelp‐forest stability in future work.

## CONCLUSION

Adding parasites to a large kelp‐forest food web made it grow into the largest published food web. This increase in network size had clear effects on scale‐dependent network metrics, most notably connectance, in contrast to predictions based on other systems. However, parasites also altered network structure in ways that were not due solely to the increase in network size. Parasites' unique life histories and trophic interaction types altered the distributions of vulnerability, generality, trophic niches, and subgraph structure of the food web, in essence changing the structure of the building blocks of the food web. Weakly connected parasites exploiting specific food chains may be an indicator of important energy flows in the ecosystem, as intermediate and upper trophic levels are most vulnerable to parasites. Parasites that can navigate the complex kelp‐forest trophic network via their life cycles are able to exploit the diverse host species that congregate in kelp forests, with many others along for the ride in transient hosts. Parasites impart unique structure on this iconic system above and beyond adding to its richness.

## CONFLICT OF INTEREST

The authors declare no conflict of interest.

## Supporting information


Appendix S1

## Data Availability

Data sets (Morton et al., 2021a) are available in Dryad: https://doi.org/10.25349/D9JG70. Code (Morton, 2021) is available in Zenodo: https://doi.org/10.5281/zenodo.5601686. The food‐web versions analyzed (Morton & Lafferty, 2021) are available in Dryad: https://doi.org/10.25349/D9Z89D.

## References

[ecm1506-bib-0001] Amundsen, P.‐A. , K. D. Lafferty , R. Knudsen , R. Primicerio , A. Klemetsen , and A. M. Kuris . 2009. “Food Web Topology and Parasites in the Pelagic Zone of a Subarctic Lake.” Journal of Animal Ecology 78: 563–72. 10.1111/j.1365-2656.2008.01518.x 19175443

[ecm1506-bib-0002] Balch, T. , and R. E. Scheibling . 2000. “Temporal and Spatial Variability in Settlement and Recruitment of Echinoderms in Kelp Beds and Barrens in Nova Scotia.” Marine Ecology Progress Series 205: 139–54.

[ecm1506-bib-0003] Banašek‐Richter, C. , M.‐F. Cattin , and L.‐F. Bersier . 2006. “Food Web Structure: From Scale Invariance to Scale Dependence, and Back Again?” In Dynamic Food Webs: Multispecies Assemblages, Ecosystem Development, and Environmental Change, edited by P. de Ruiter, V. Wolters, J. C. Moore and K. Melville‐Smith, 48–55. Cambridge: Academic Press.

[ecm1506-bib-0004] Beas‐Luna, R. , M. Novak , M. H. Carr , M. T. Tinker , A. Black , J. E. Caselle , M. Hoban , D. Malone , and A. Iles . 2014. “An Online Database for Informing Ecological Network Models: http://kelpforest.ucsc.edu.” PLoS One 9: e109356. 10.1371/journal.pone.0109356 25343723 PMC4208745

[ecm1506-bib-0005] Benesh, D. P. , G. A. Parker , J. C. Chubb , and K. D. Lafferty . 2020. “Trade‐Offs with Growth Limit Host Range in Complex Life‐Cycle Helminths.” American Naturalist 197: E000–E54. 10.1086/712249 33523790

[ecm1506-bib-0006] Bethel, W. M. , and J. C. Holmes . 1977. “Increased Vulnerability of Amphipods to Predation Owing to Altered Behavior Induced by Larval Acanthocephalans.” Canadian Journal of Zoology 55: 110–5. 10.1139/z77-013 837268

[ecm1506-bib-0007] Byrnes, J. E. , D. C. Reed , B. J. Cardinale , K. C. Cavanaugh , S. J. Holbrook , and R. J. Schmitt . 2011. “Climate‐Driven Increases in Storm Frequency Simplify Kelp Forest Food Webs.” Global Change Biology 17: 2513–24. 10.1111/j.1365-2486.2011.02409.x

[ecm1506-bib-0008] Carr, M. H. , and D. C. Reed . 2016. “Shallow Rocky Reefs and Kelp Forests.” In Ecosystems of California, edited by H. Mooney and E. Zavaleta, 311–36. Berkeley, CA: University of California Press.

[ecm1506-bib-0009] Cerda, O. , I. A. Hinojosa , and M. Thiel . 2010. “Nest‐Building Behavior by the Amphipod Peramphithoe Femorata (Krøyer) on the Kelp *Macrocystis pyrifera* (Linnaeus) C. Agardh from Northern‐Central Chile.” Biological Bulletin 218: 248–58. 20570848 10.1086/BBLv218n3p248

[ecm1506-bib-0010] Chen, H. , K. Shao , C. W. Liu , W. Lin , and W. Liu . 2011. “The Reduction of Food Web Robustness by Parasitism: Fact and Artefact.” International Journal for Parasitology 41: 627–34. 10.1016/j.ijpara.2010.12.013 21296081

[ecm1506-bib-0011] Combes, C. 2001. Parasitism: The Ecology and Evolution of Intimate Interactions. Chicago, IL: University of Chicago Press.

[ecm1506-bib-0012] Coyer, J. A. 1984. “The Invertebrate Assemblage Associated with the Giant Kelp, *Macrocystis Pyrifera*, at Santa Catalina Island, California: A General Description with Emphasis on Amphipods, Copepods, Mysids, and Shrimps.” Fishery Bulletin 82: 55–66.

[ecm1506-bib-0013] Csardi, G. , and T. Nepusz . 2006. “The Igraph Software Package for Complex Network Research.” InterJournal, Complex Systems 1695: 1–9.

[ecm1506-bib-0014] Dayton, P. K. 1985. “Ecology of Kelp Communities.” Annual Review of Ecology and Systematics 16: 215–45.

[ecm1506-bib-0015] Dayton, P. K. , and M. J. Tegner . 1984. “Catastrophic Storms, El Niño, and Patch Stability in a Southern California Kelp Community.” Science 224: 283–5. 17734914 10.1126/science.224.4646.283

[ecm1506-bib-0016] Dunne, J. , R. Williams , and N. Martinez . 2004. “Network Structure and Robustness of Marine Food Webs.” Marine Ecology Progress Series 273: 291–302. 10.3354/meps273291

[ecm1506-bib-0017] Dunne, J. A. 2006. “The Network Structure of Food Webs.” In Ecological Networks: Linking Structure to Dynamics in Food Webs, edited by M. Pascual and J.A. Dunne , 27–86. Oxford: Oxford University Press.

[ecm1506-bib-0018] Dunne, J. A. , K. D. Lafferty , A. P. Dobson , R. F. Hechinger , A. M. Kuris , N. D. Martinez , J. P. McLaughlin , et al. 2013. “Parasites Affect Food Web Structure Primarily through Increased Diversity and Complexity.” PLoS Biology 11: e1001579. 10.1371/journal.pbio.1001579 23776404 PMC3679000

[ecm1506-bib-0019] Dunne, J. A. , R. J. Williams , and N. D. Martinez . 2002. “Food‐Web Structure and Network Theory: The Role of Connectance and Size.” Proceedings of the National Academy of Sciences USA 99: 12917–22. 10.1073/pnas.192407699 PMC130560 12235364

[ecm1506-bib-0020] Ebeling, A. W. , D. R. Laur , and R. J. Rowley . 1985. “Severe Storm Disturbances and Reversal of Community Structure in a Southern California Kelp Forest.” Marine Biology 84: 287–94. 10.1007/BF00392498

[ecm1506-bib-0021] Edwards, M. S. 2004. “Estimating Scale‐Dependency in Disturbance Impacts: El Niños and Giant Kelp Forests in the Northeast Pacific.” Oecologia 138: 436–47. 10.1007/s00442-003-1452-8 14673640

[ecm1506-bib-0022] Goldwasser, L. , and J. Roughgarden . 1997. “Sampling Effects and the Estimation of Food‐Web Properties.” Ecology 78: 41–54.

[ecm1506-bib-0023] Graham, M. , B. Halpern , M. Carr , T. R. McClanahan , and G. R. Branch . 2008. “Diversity and Dynamics of California Subtidal Kelp Forests.” In Food Webs and the Dynamics of Marine Reefs, edited by T.R. McLanahan and G.R. Branch , 103–34. Oxford: Oxford University Press.

[ecm1506-bib-0024] Graham, M. H. 2004. “Effects of Local Deforestation on the Diversity and Structure of Southern California Giant Kelp Forest Webs.” Ecosystems 7: 341–57. 10.1007/s10021-003-0245-6

[ecm1506-bib-0025] Hall, S. J. , and D. G. Raffaelli . 1993. “Food Webs: Theory and Reality.” Advances in Ecological Research 24: 187–239. 10.1016/S0065-2504(08)60043-4

[ecm1506-bib-0026] Hechinger, R. F. , and K. D. Lafferty . 2005. “Host Diversity Begets Parasite Diversity: Bird Final Hosts and Trematodes in Snail Intermediate Hosts.” Proceedings of the Royal Society B: Biological Sciences 272: 1059–66. 10.1098/rspb.2005.3070 PMC1599879 16024365

[ecm1506-bib-0027] Hechinger, R. F. , K. D. Lafferty , A. P. Dobson , J. H. Brown , and A. M. Kuris . 2011. “A Common Scaling Rule for Abundance, Energetics, and Production of Parasitic and Free‐Living Species.” Science 333: 445–8. 21778398 10.1126/science.1204337 PMC3236646

[ecm1506-bib-0028] Hobson, E. S. , and J. R. Chess . 1976. “Trophic Interactions among Fishes and Zooplankters near Shore at Santa Catalina Island, California.” Fishery Bulletin 74: 567.

[ecm1506-bib-0029] Hudson, L. N. , R. Emerson , G. B. Jenkins , K. Layer , M. E. Ledger , D. E. Pichler , M. S. A. Thompson , E. J. O'Gorman , G. Woodward , and D. C. Reuman . 2013. “Cheddar: Analysis and Visualisation of Ecological Communities in R.” Methods in Ecology and Evolution 4: 99–104. 10.1111/2041-210X.12005

[ecm1506-bib-0030] Johnson, P. T. J. , A. Dobson , K. D. Lafferty , D. J. Marcogliese , J. Memmott , S. A. Orlofske , R. Poulin , and D. W. Thieltges . 2010. “When Parasites Become Prey: Ecological and Epidemiological Significance of Eating Parasites.” Trends in Ecology & Evolution 25: 362–71. 10.1016/j.tree.2010.01.005 20185202

[ecm1506-bib-0031] Johnson, S. , V. Domínguez‐García , L. Donetti , and M. A. Muñoz . 2014. “Trophic Coherence Determines Food‐Web Stability.” Proceedings of the National Academy of Science USA 111: 17923–8. 10.1073/pnas.1409077111 PMC4273378 25468963

[ecm1506-bib-0032] Køie, M. 1993. “Aspects of the Life Cycle and Morphology of *Hysterothylacium Aduncum* (Rudolphi, 1802) (Nematoda, Ascaridoidea, Anisakidae).” Canadian Journal of Zoology 71: 1289–96.

[ecm1506-bib-0033] Kones, J. K. , K. Soetaert , D. van Oevelen , and J. O. Owino . 2009. “Are Network Indices Robust Indicators of Food Web Functioning? A Monte Carlo Approach.” Ecological Modelling 220: 370–82. 10.1016/j.ecolmodel.2008.10.012

[ecm1506-bib-0034] Kuris, A. M. , R. F. Hechinger , J. C. Shaw , K. L. Whitney , L. Aguirre‐Macedo , C. A. Boch , A. P. Dobson , et al. 2008. “Ecosystem Energetic Implications of Parasite and Free‐Living Biomass in Three Estuaries.” Nature 454: 515–8. 10.1038/nature06970 18650923

[ecm1506-bib-0035] Kushner, D. J. , A. Rassweiler , J. P. McLaughlin , and K. D. Lafferty . 2013. “A Multi‐Decade Time Series of Kelp Forest Community Structure at the California Channel Islands: Ecological Archives E094‐245.” Ecology 94: 2655–5.

[ecm1506-bib-0036] Lafferty, K. D. 2012. “Biodiversity Loss Decreases Parasite Diversity: Theory and Patterns.” Philosophical Transactions of the Royal Society B 367: 2814–27. 10.1098/rstb.2012.0110 PMC3427564 22966137

[ecm1506-bib-0037] Lafferty, K. D. , S. Allesina , M. Arim , C. J. Briggs , G. De Leo , A. P. Dobson , J. Dunne , et al. 2008. “Parasites in Food Webs: The Ultimate Missing Links.” Ecology Letters 11: 533–46. 10.1111/j.1461-0248.2008.01174.x 18462196 PMC2408649

[ecm1506-bib-0038] Lafferty, K. D. , A. P. Dobson , and A. M. Kuris . 2006. “Parasites Dominate Food Web Links.” Proceedings of the National Academy of Sciences USA 103: 11211–6. 10.1073/pnas.0604755103 PMC1544067 16844774

[ecm1506-bib-0039] Lafferty, K. D. , and A. M. Kuris . 2009. “Parasites Reduce Food Web Robustness because they Are Sensitive to Secondary Extinction as Illustrated by an Invasive Estuarine Snail.” Philosophical Transactions of the Royal Society of London, Series B, Biological sciences 364: 1659–63. 10.1098/rstb.2008.0220 19451117 PMC2685421

[ecm1506-bib-0040] Lafferty, K. D. , and A. M. Kuris . 2002. “Trophic Strategies, Animal Diversity and Body Size.” Trends in Ecology & Evolution 17: 507–13. 10.1016/S0169-5347(02)02615-0

[ecm1506-bib-0041] Lafferty, K. D. , and A. K. Morris . 1996. “Altered Behavior of Parasitized Killifish Increases Susceptibility to Predation by Bird Final Hosts.” Ecology 77: 1390–7. 10.2307/2265536

[ecm1506-bib-0042] Leung, T. L. F. , D. B. Keeney , and R. Poulin . 2009. “Cryptic Species Complexes in Manipulative Echinostomatid Trematodes: When Two Become Six.” Parasitology 136: 241–52. 19091157 10.1017/S0031182008005374

[ecm1506-bib-0043] Marcogliese, D. J. 1996. “Transmission of the Sealworm, *Pseudoterranova Decipiens* (Krabbe), from Invertebrates to Fish in an Enclosed Brackish Pond.” Journal of Experimental Marine Biology and Ecology 205: 205–19.

[ecm1506-bib-0044] Martinez, N. D. 1994. “Scale‐Dependent Constraints on Food‐Web Structure.” American Naturalist 144: 935–53.

[ecm1506-bib-0045] Martinez, N. D. 1993. “Effects of Resolution on Food Web Structure.” Oikos 66: 403–12.

[ecm1506-bib-0046] Martinez, N. D. , and J. H. Lawton . 1995. “Scale and Food‐Web Structure: From Local to Global.” Oikos 73: 148–54. 10.2307/3545903

[ecm1506-bib-0047] McLaughlin, J. P. 2018. The Food Web for the Sand Flats at Palmyra Atoll. Santa Barbara, CA: University of California.

[ecm1506-bib-0048] McLaughlin, J. P. , D. N. Morton , and K. D. Lafferty . 2020. “Parasites in Marine Food Webs.” In Marine Disease Ecology, edited by D. C. Behringer, B. R. Silliman and K. D. Lafferty, 45. Oxford: Oxford University Press.

[ecm1506-bib-0049] Milo, R. , S. Shen‐Orr , S. Itzkovitz , N. Kashtan , D. Chklovskii , and U. Alon . 2002. “Network Motifs: Simple Building Blocks of Complex Networks.” Science 298: 824–7. 10.1126/science.298.5594.824 12399590

[ecm1506-bib-0050] Miura, O. , A. M. Kuris , M. E. Torchin , R. F. Hechinger , E. J. Dunham , and S. Chiba . 2005. “Molecular‐Genetic Analyses Reveal Cryptic Species of Trematodes in the Intertidal Gastropod, *Batillaria cumingi* (Crosse).” International Journal for Parasitology 35: 793–801. 15925598 10.1016/j.ijpara.2005.02.014

[ecm1506-bib-0051] Morton, D. N. 2020. The Effects of Parasites on the Kelp‐Forest Food Web. Santa Barbara, CA: University of California.

[ecm1506-bib-0052] Morton, D. N. , C. Antonino , F. Broughton , L. Dykman , A. Kuris , and K. Lafferty . 2021a. “Data from: A Food Web Including Parasites for Kelp Forests of the Santa Barbara Channel, California.” Dryad Digital Repository 3: 99. 10.25349/D9JG70 PMC8032823 33833244

[ecm1506-bib-0053] Morton, D. N. , C. Y. Antonino , F. J. Broughton , L. N. Dykman , A. M. Kuris , and K. D. Lafferty . 2021b. “A Food Web Including Parasites for Kelp Forests of the Santa Barbara Channel, California.” Scientific Data 8: 99. 10.1038/s41597-021-00880-4 33833244 PMC8032823

[ecm1506-bib-0054] Morton, D. . 2021. “dananmorton/Kelp.food.web.Morton: Species.level.food.webs (v1.0.0).” *Zenodo*. 10.5281/zenodo.5601686.

[ecm1506-bib-0055] Morton, D. , and K. Lafferty . 2021. “Parasites in Kelp‐Forest Food Webs Increase Food‐Chain Length, Complexity, and Specialization, but Reduce Connectance.” *Dryad*, dataset. 10.25349/D9Z89D. PMC9286845 35865510

[ecm1506-bib-0056] Mouritsen, K. N. , and R. Poulin . 2002. “Parasitism, Community Structure and Biodiversity in Intertidal Ecosystems.” Parasitology 124: 101–17. 10.1017/S0031182002001476 12396219

[ecm1506-bib-0057] Ness, J. H. , and S. A. Foster . 1999. “Parasite‐Associated Phenotype Modifications in Threespine Stickleback.” Oikos 85: 127. 10.2307/3546798

[ecm1506-bib-0058] Paine, R. T. 1988. “Food Webs: Road Maps of Interactions or Grist for Theoretical Development?” Ecology 69: 1648–54.

[ecm1506-bib-0059] Palm, H. W. , and J. N. Caira . 2008. “Host Specificity of Adult Versus Larval Cestodes of the Elasmobranch Tapeworm Order Trypanorhyncha.” International Journal for Parasitology 38: 381–8. 10.1016/j.ijpara.2007.08.011 17950740

[ecm1506-bib-0060] Parker, G. A. , J. C. Chubb , M. A. Ball , and G. N. Roberts . 2003. “Evolution of Complex Life Cycles in Helminth Parasites.” Nature 425: 480–4. 10.1038/nature02012 14523438

[ecm1506-bib-0061] Polis, G. A. 1991. “Complex Trophic Interactions in Deserts: An Empirical Critique of Food‐Web Theory.” American Naturalist 138: 123–55. 10.1086/285208

[ecm1506-bib-0062] Poulin, R. 2011. “Uneven Distribution of Cryptic Diversity among Higher Taxa of Parasitic Worms.” Biology Letters 7: 241–4. 10.1098/rsbl.2010.0640 20861036 PMC3061155

[ecm1506-bib-0063] R Core Team . 2019. R: A Language and Environment for Statistical Computing. Vienna: R Foundation for Statistical Computing.

[ecm1506-bib-0064] Reide, J. O. , B. C. Rall , C. Banasek‐Richter , S. A. Navarrete , E. A. Wieters , M. C. Emmerson , U. Jacob , and U. Brose . 2010. “Scaling of Food‐Web Properties with Diversity and Complexity across Ecosystems.” In Advances in Ecological Research, edited by G. Woodward, 42: 139–170. Amsterdam: Elsevier. 10.1016/B978-0-12-381363-3.00003-4

[ecm1506-bib-0065] Rogers‐Bennett, L. , and C. A. Catton . 2019. “Marine Heat Wave and Multiple Stressors Tip Bull Kelp Forest to Sea Urchin Barrens.” Scientific Reports 9: 1–9. 10.1038/s41598-019-51114-y 31636286 PMC6803666

[ecm1506-bib-0066] Romanuk, T. N. , Y. Zhou , F. S. Valdovinos , and N. D. Martinez . 2017. “Robustness Trade‐Offs in Model Food Webs: Invasion Probability Decreases while Invasion Consequences Increase with Connectance.” In Advances in Ecological Research, Networks of Invasion: A Synthesis of Concepts, edited by D.A. Bohan , A.J. Dumbrell , and F. Massol , 263–91. Cambridge: Academic Press. 10.1016/bs.aecr.2016.11.001

[ecm1506-bib-0067] Rudolf, V. H. W. , and K. D. Lafferty . 2011. “Stage Structure Alters how Complexity Affects Stability of Ecological Networks: Stage Structure and Network Stability.” Ecology Letters 14: 75–9. 10.1111/j.1461-0248.2010.01558.x 21114747

[ecm1506-bib-0068] Schiel, D. R. , and M. S. Foster . 2015. The Biology and Ecology of Giant Kelp Forests. Berkeley, CA: University of California Press.

[ecm1506-bib-0069] Schoener, T. W. 1989. “Food Webs from the Small to the Large: The Robert H. MacArthur Award Lecture.” Ecology 70: 1559–89. 10.2307/1938088

[ecm1506-bib-0070] Soldánová, M. , S. Georgieva , J. Roháčová , R. Knudsen , J. A. Kuhn , E. H. Henriksen , A. Siwertsson , et al. 2017. “Molecular Analyses Reveal High Species Diversity of Trematodes in a Sub‐Arctic Lake.” International Journal for Parasitology 47: 327–45. 10.1016/j.ijpara.2016.12.008 28315362

[ecm1506-bib-0071] Stouffer, D. B. , J. Camacho , and L. A. N. Amaral . 2006. “A Robust Measure of Food Web Intervality.” Proceedings of the National Academy of Sciences USA 103: 19015–20. 10.1073/pnas.0603844103 PMC1748169 17146055

[ecm1506-bib-0072] Thompson, R. M. , K. N. Mouritsen , and R. Poulin . 2005. “Importance of Parasites and their Life Cycle Characteristics in Determining the Structure of a Large Marine Food Web.” Journal of Animal Ecology 74: 77–85. 10.1111/j.1365-2656.2004.00899.x

[ecm1506-bib-0073] Vinagre, C. , M. Costa , S. Wood , R. Williams , and J. Dunne . 2019. “Potential Impacts of Climate Change and Humans on the Trophic Network Organization of Estuarine Food Webs.” Marine Ecology Progress Series 616: 13–24. 10.3354/meps12932

[ecm1506-bib-0074] Warren, C. P. , M. Pascual , K. D. Lafferty , and A. M. Kuris . 2010. “The Inverse Niche Model for Food Webs with Parasites.” Theoretical Ecology 3: 285–94. 10.1007/s12080-009-0069-x 25540673 PMC4270433

[ecm1506-bib-0075] Warren, P. H. 1990. “Variation in Food‐Web Structure: The Determinants of Connectance.” American Naturalist 136: 689–700. 10.1086/285123

[ecm1506-bib-0076] Williams, R. J. , and N. D. Martinez . 2008. “Success and its Limits among Structural Models of Complex Food Webs.” Journal of Animal Ecology 77: 512–9. 10.1111/j.1365-2656.2008.01362.x 18284474

[ecm1506-bib-0077] Williams, R. J. , and N. D. Martinez . 2000. “Simple Rules Yield Complex Food Webs.” Nature 404: 180–3. 10.1038/35004572 10724169

[ecm1506-bib-0078] Williams, R. J. , and D. W. Purves . 2011. “The Probabilistic Niche Model Reveals Substantial Variation in the Niche Structure of Empirical Food Webs.” Ecology 92: 1849–57. 10.1890/11-0200.1 21939081

[ecm1506-bib-0079] Winemiller, K. O. , and G. A. Polis . 1996. “Food Webs: What Can they Tell us about the World?” In Food Webs: Integration of Patterns & Dynamics, edited by G.A. Polis and K.O. Winemiller , 1–22. Boston, MA: Springer US. 10.1007/978-1-4615-7007-3_1

[ecm1506-bib-0080] Wood, S. A. , R. Russell , D. Hanson , R. J. Williams , and J. A. Dunne . 2015. “Effects of Spatial Scale of Sampling on Food Web Structure.” Ecology and Evolution 5: 3769–82. 10.1002/ece3.1640 26380704 PMC4567879

[ecm1506-bib-0081] Zuercher, R. , and A. W. E. Galloway . 2019. “Coastal Marine Ecosystem Connectivity: Pelagic Ocean to Kelp Forest Subsidies.” Ecosphere 10: e02602. 10.1002/ecs2.2602

