# Supplementary material for: Parasites in kelp‐forest food webs increase food‐chain length, complexity, and specialization, but reduce connectance
Source: Ecol Monogr. 2022 Mar 7;92(2):e1506. doi: 10.1002/ecm.1506 (PMC9286845; doi:10.1002/ecm.1506)

Supporting Information. Morton DN, Lafferty KD. Parasites in kelp-forest food webs increase food-chain length, complexity, and specialization, but reduce connectance. Ecological Monographs

Appendix S1

Table S1. Model errors for the motif frequencies. Positive MEs indicate a motif was over-represented in the empirical food web relative to the niche model. |ME|> 1 indicate that empirical values were significantly different from model predictions. If empirical webs have different (different >1) MEs, this indicates the frequency of that motif varies between the webs.

	D1	D2	D3	D4	D5	D6	D7	D8	S1	S2	S3	S4	S5
Predator-Prey	2.30	4.47	6.63	11.92	7.57	10.83	3.31	7.38	0.26	0.58	39.75	2.65	0.42
Predator-Prey + Parasite-Host	1.43	10.84	4.97	26.25	14.71	11.30	4.81	9.78	-2.09	0.05	71.76	-1.54	-2.83
Predator-Prey + Parasite-Host + Predator-Parasite	3.77	12.38	27.62	71.91	120.87	31.67	25.77	111.30	-0.77	1.92	1225.90	-1.72	1.63

Figures

Figure S1. Graphs of each subweb in the kelp-forest food web. Blue nodes are free-living taxa, red nodes are parasites, green are autotrophs, and brown are detritus. Each node represents a species, and arrows point from consumers to resources. (a) predator-prey web, with links in blue (b) parasite-host web, with links in red, and (c) predator-parasite web, with links in orange. All nodes are in the same position on each plot, with the network arranged vertically by prey-averaged trophic level (horizontal arrangement is random), so top consumers are at the top and producers and detritus are at the bottom. Some nodes may be obscured due to the size of the network. Created in *igraph* (Csardi and Nepusz, 2006) in R Version 3.6.2 (R Core Team 2019).

Figures S2-S7. Graphical representation of each version of the food web as a matrix, with species ordered to minimize the number of gaps in the diet (simulated annealing method, Stouffer et al. 2006). Consumers are along the x-axis, resources along the y-axis. Matrices are identical in scale, but the order of species may vary among plots. Points indicate trophic links: blue are predator-prey (free-living) interactions, red are parasite-host interactions, and black are predator-parasite interactions (cases where a predator consumed a parasite, leading to the death of the parasite). The vertical spread of points over a consumer indicates its feeding niche. See below for data sources.

Figures S2-S4. Data from: Hechinger, Ryan F., Kevin D. Lafferty, John P. McLaughlin, Brian L. Fredensborg, Todd C. Huspeni, Julio Lorda, Parwant K. Sandhu, et al. "Food Webs Including Parasites, Biomass, Body Sizes, and Life Stages for Three California/Baja California Estuaries." Ecology 92, no. 3 (2011): 791–791. https://doi.org/10.1890/10-1383.1.

Figure S5. Data from: Zander, C. Dieter, Neri Josten, Kim C. Detloff, Robert Poulin, John P. McLaughlin, and David W. Thieltges. “Food Web Including Metazoan Parasites for a Brackish Shallow Water Ecosystem in Germany and Denmark.” Ecology 92, no. 10 (2011): 2007–2007. https://doi.org/10.1890/11-0374.1.

Figure S6. Data from: Mouritsen, Kim N., Robert Poulin, John P. McLaughlin, and David W. Thieltges. “Food Web Including Metazoan Parasites for an Intertidal Ecosystem in New Zealand.” Ecology 92, no. 10 (2011): 2006–2006. https://doi.org/10.1890/11-0371.1.

Figure S7. Data from: Thieltges, David W., Karsten Reise, Kim N. Mouritsen, John P. McLaughlin, and Robert Poulin. “Food Web Including Metazoan Parasites for a Tidal Basin in Germany and Denmark.” Ecology 92, no. 10 (2011): 2005–2005. https://doi.org/10.1890/11-0351.1.

Literature Cited

Csardi, G., Nepusz, T., 2006. The *igraph* software package for complex network research. InterJournal, Complex Systems 1695, 1–9.

R Core Team. 2019. R: A Language and Environment for Statistical Computing. Vienna: R Foundation for Statistical Computing.

Stouffer, D. B., J. Camacho, and L. A. N. Amaral. 2006. “A Robust Measure of Food Web Intervality.” Proceedings of the National Academy of Sciences USA 103: 19015–20. https://doi.org/10.1073/pnas.0603844103

Figure S1.

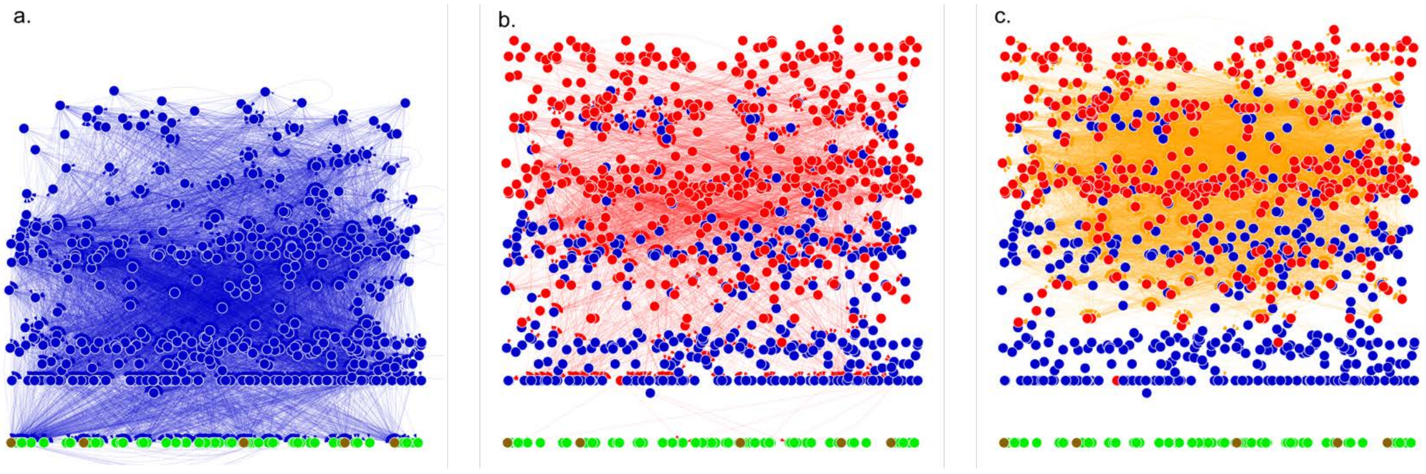

Figure S2. BSQ niche plots

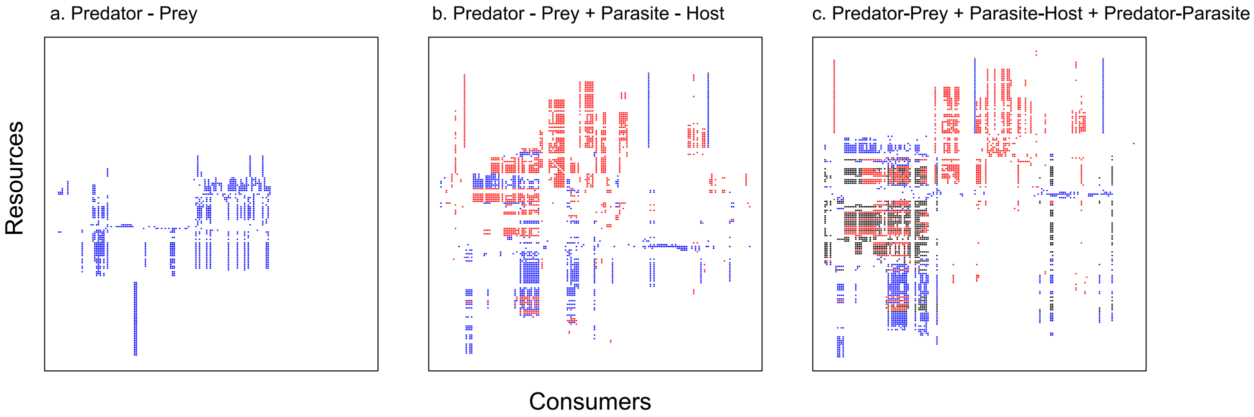

Figure S3. CSM niche plots

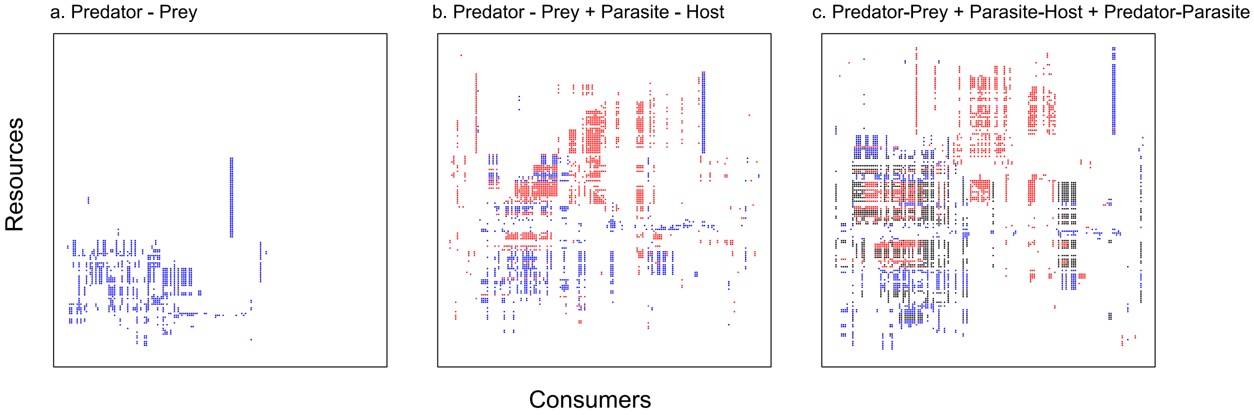

Figure S4. EPB niche plots

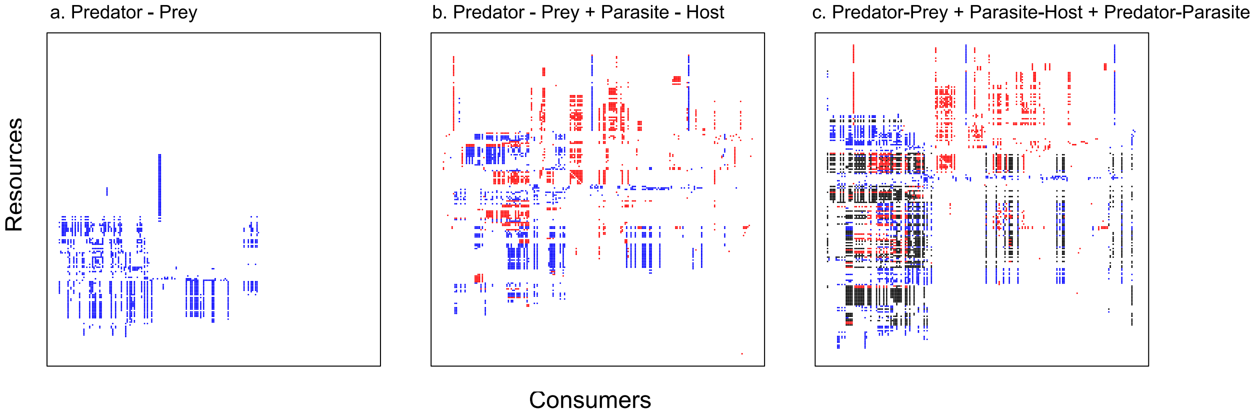

Figure S5. Flensburg niche plots

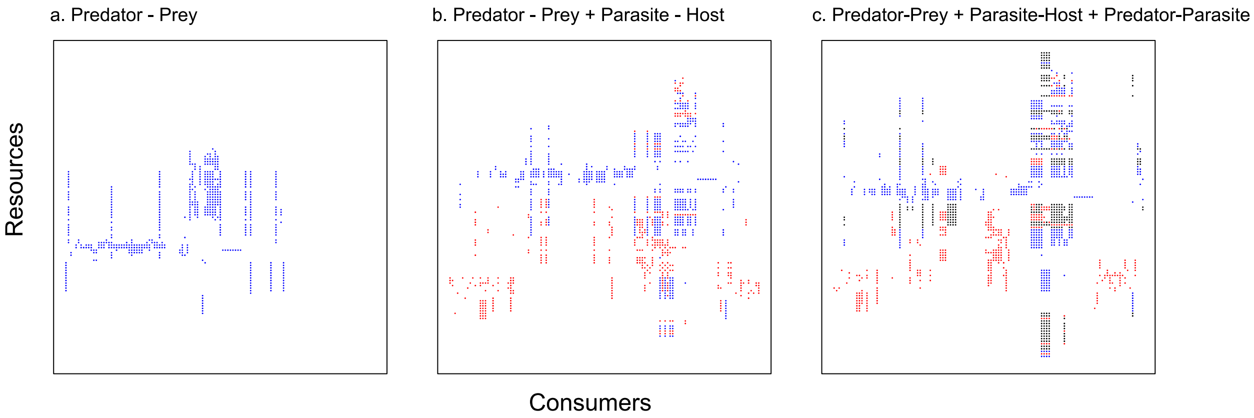

Figure S6. Otago niche plots

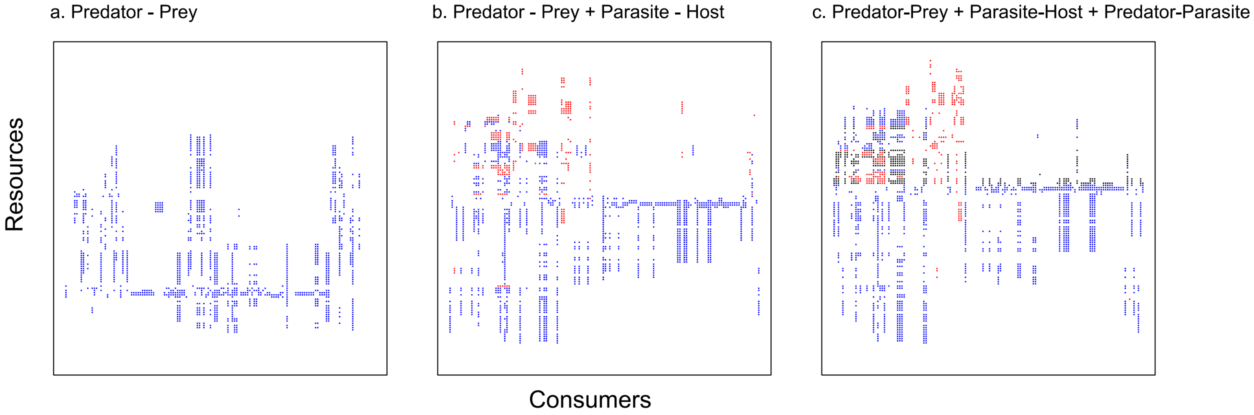

Figure S7. Sylt niche plots

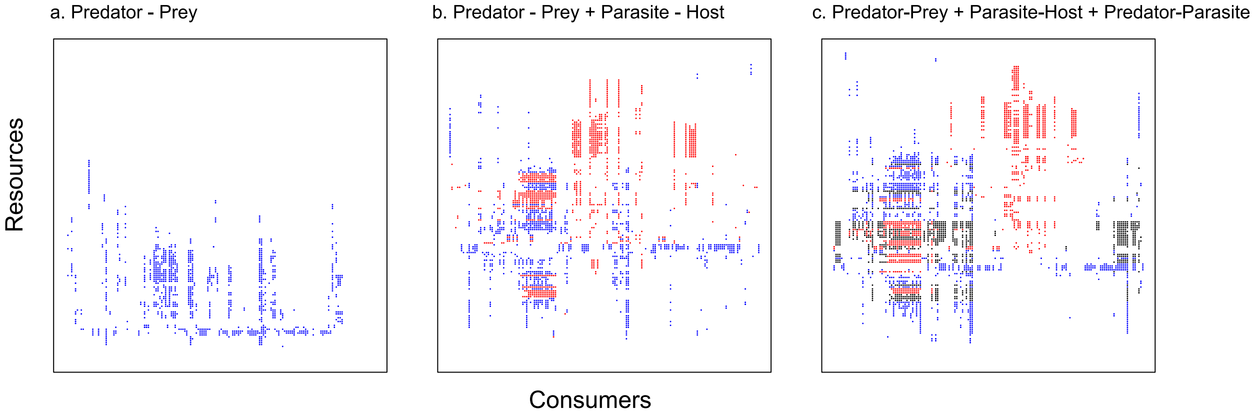

Supplement: Supplementary file 1 — Appendix S1 [file ECM-92-0-s001.pdf]
